# Filamentous nuclear actin regulation of PML NBs during the DNA damage response is deregulated by prelamin A

**DOI:** 10.1038/s41419-022-05491-4

**Published:** 2022-12-15

**Authors:** Andrew M. Cobb, Shanelle A. De Silva, Robert Hayward, Karolina Sek, Svenja Ulferts, Robert Grosse, Catherine M. Shanahan

**Affiliations:** 1 BHF Centre of Research Excellence, School of Cardiovascular Medicine and Sciences, King’s College London, The James Black Centre, 125 Coldharbour Lane, London, SE5 9NU United Kingdom; 2 Institute of Experimental and Clinical Pharmacology and Toxicology, Medical Faculty, University of Freiburg, Albertstraße 25, 79104 Freiburg, Germany

**Keywords:** Cell biology, Nucleoskeleton

## Abstract

Nuclear actin participates in a continuously expanding list of core processes within eukaryotic nuclei, including the maintenance of genomic integrity. In response to DNA damage, nuclear actin polymerises into filaments that are involved in the repair of damaged DNA through incompletely defined mechanisms. We present data to show that the formation of nuclear F-actin in response to genotoxic stress acts as a scaffold for PML NBs and that these filamentous networks are essential for PML NB fission and recruitment of microbodies to DNA lesions. Further to this, we demonstrate that the accumulation of the toxic lamin A precursor prelamin A induces mislocalisation of nuclear actin to the nuclear envelope and prevents the establishment of nucleoplasmic F-actin networks in response to stress. Consequently, PML NB dynamics and recruitment to DNA lesions is ablated, resulting in impaired DNA damage repair. Inhibition of nuclear export of formin mDia2 restores nuclear F-actin formation by augmenting polymerisation of nuclear actin in response to stress and rescues PML NB localisation to sites of DNA repair, leading to reduced levels of DNA damage.

## Introduction

Actin is a fundamental cytoskeletal component of eukaryotic cells that orchestrates cell shape, motility and molecular transport—all of which require the spatiotemporal polymerisation of globular monomeric actin into filamentous actin (F-actin) [1]. Advances in molecular imaging technologies has led to the discovery that actin is also present in the nucleus, and that it is able to form transient F-actin networks in response to specific environmental cues [2–5].

Mitotic exit [6], cell spreading [3] and the serum response [7] are recognised to drive nuclear actin polymerisation. Alongside these, cellular stressors, including heat shock [8, 9], ATP depletion [10], oxidative stress [11], DNA damage [12–15] and other exogenous toxic agents [16] have also been shown to be principal inducers of nuclear actin filament formation. Studies have shown that nuclear actin is important for essential cell functions such as DNA replication [17] and transcription [18, 19] and it has been implicated in several diseases ranging from cancer [20] to neurodegeneration [21, 22]. Thus, it is evident that actin participates in critical activities within nuclei that are necessary for key cellular functions and for human health.

The maintenance of genome stability is a vital process that involves a vast array of diverse factors and signalling networks that have evolved to protect cells from aberrant genetic alterations. Lesions in DNA are detected by surveillance complexes [23] that instigate DNA damage response (DDR) pathways to enable damage removal in a substrate-dependent manner [24]. Whilst much is known about specific repair factors and signalling components, the underlying mechanisms that determine the temporal dynamics and movement of these components during the DDR are less well established.

Recently, nuclear F-actin has been shown to participate in the DDR in the retention of Ku70/80 at DNA double-strand breaks (DSBs) [25], DSB clearance [12], WNT-mediated regulation of the DDR [15], ATR-recruitment during single-strand break repair [26] and also an advocated function in stalled replication fork repair [27]. Strong evidence shows that nuclear F-actin arises in response to DSBs and facilitates homologous recombination via mediating chromatin dynamics [13] and DNA break clustering [14]. Whilst the majority of findings implicate nuclear F-actin in DSB repair, it’s noteworthy that DNA damaging agents that cause other types of DNA damage, such as pyramidine dimers or oxidised base derivates [12], also stimulate nuclear F-actin accrual—suggesting the function of nuclear actin in DNA repair is not restricted to resolving DSBs alone.

One component of the DDR whose precise activity has remained somewhat elusive is the promyelocytic leukaemia (PML) nuclear body (NB). This subnuclear compartment is a tumour suppressor [28] that acts as a DNA damage sensor [29] and as a hub for DSB repair proteins [30, 31]. PML NBs also activate p53 in response to stress [32] and associate with DDR kinases during the DDR [33]. Induction of DNA damage causes PML NBs to increase in number by a supramolecular fission that is intrinsic to the DDR. These microbodies localise to sites of DNA repair and facilitate the resolution of damage [29, 34, 35]. PML NB fission in response to DNA damage requires SUMO-1 activity [35] alongside DDR proteins NBS1, ATM, Chk2 and ATR [29]. Interestingly, it is thought there are non-arbitrary pre-determined locations for PML microbodies prior to their formation [35], but what determines this localisation is not known.

Another important factor for efficient DNA repair is an intact and functional nuclear envelope (NE). In particular, the accumulation of toxic non-mature lamins—including prelamin A—are detrimental to the DDR and results in genomic instability [36–38]. Although it has been established that a dysregulated NE or compromised nuclear lamina can impinge on genomic integrity through various mechanisms, it has not been shown if these maladaptations affect nuclear F-actin and its activity during the DDR. Importantly, in vitro assays have demonstrated that nuclear actin interacts with A-type [39, 40] and B-type lamins [40] as well as with the NE protein Emerin [41], suggesting F-actin function is likely to be influenced by changes to the NE.

We wanted to further characterise the role of nuclear F-actin in the DDR and to understand if detrimental changes to the NE that are associated with ageing affected this activity. Using recently developed nuclear actin-specific probes, we show that F-actin forms in response to different types of DNA lesions in U2OS cells and that depletion of nuclear actin pools cause increased DNA damage and reduced cell vitality. We also show that expression of prelamin A causes marked alterations to nuclear actin, with loss of F-actin formation in response to stress and accumulation of actin at the NE. In addition, we provide evidence that the DDR protein PML localises along F-actin filaments, but this positioning and subsequent DDR-related function of PML is attenuated by prelamin A. Lastly, we show that F-actin networks can be partially restored in prelamin A expressing cells using Leptomycin B and when used in combination with Remodelin can significantly reduce the genotoxic properties of prelamin A.

## Materials and methods

### Cells/treatments

Osteosarcoma cells (U2OS) were obtained from American Tissue Culture Collection. Cells were passaged at 70% confluency and maintained in DMEM complete media (Sigma) supplemented with 10 units/mL penicillin, 10 mg/mL streptomycin, 200 mM l-glutamine and 10% FBS (or 0.5% FBS during serum starvation experiments).

U2OS cells stably expressing the nuclear actin chromobody were generated by lentiviral transduction. HEK293T cells grown on 10 cm dishes were simultaneously transfected with pWPXL-AC-tagGFP-NLS as well as the packaging vector psPAX2 and the envelope plasmid pMD2.G. The supernatant containing viral particles was harvested 48 h post transfection by filtering through a 0.45 µm sterile filter. U2OS cells were transduced with medium containing viral particles in six-well plates and replaced with fresh medium after 72 h. Targeted U2OS cells were cultured for four passages before sorting for GFP-positive cells using the BD FACSMelody system.

DNA damage treatments: For DSB DNA damage induction, cells were typically treated for 3 h with 1 mM etoposide. H_2_O_2_ (Sigma) was used at 200 µM for 3 h or Neocarzinostatin (NCS) at 0.8 µg/ml for 2 h to induce oxidative DNA damage. For bulky DNA adducts, UV irradiation was performed using a UV Stratalinker 1800 (Stratagene), typically at 50 J/m^2^ unless stated. For localised UV damage, Isopore 0.5 mm membrane filters (Merck, Darmstadt, Germany) were used.

Non-DNA damage cell treatments: Farnesyltransferase inhibitor FTI-276 (R&D Systems, Minneapolis, USA) was used at 25 µM and Remodelin (R&D Systems) was used at 10 µM. Both treatments were for 48 h. Retinoic acid receptor (RAR) agonist AC261066 and RAR antagonist BMS453 (both Tocris, Oxford, UK) were used for 16 h at 1 µM. Leptomycin B (LMB) (Cell Signalling Technology, Danvers, USA) was used at 20 nM for 0.5 or 2 h.

### Adenoviral constructs and transfections

U2OS cells at 70% confluence were infected with adenovirus containing a FLAG-tagged uncleavable form of prelamin A mutated within the Zmpste24/Face1 cleavage site (L647R) (Prelamin A), wild-type mature lamin A (WTLA) or EGFP. Adenovirus containing mCherry-EXP6, mCherry-PML (VectorBuilder, Chicago, USA) or KASH (gift from Dr.Qiuping Zhang) were also used. The multiplicity of infection was 5 particles per cell, routinely achieving >80% transduction efficiency as assessed by the control EGFP.

Nuclear actin Chromobody TagGFP2 (ChromoTek, Munich, Germany)—hereafter referred to as GFP-nAC—or GFP control (GFP) was transfected into U2OS cells at 70% confluency using Viafect (Promega, Madison, USA). For small interfering RNA–mediated interference of Face1, ON-TARGETplus Human ZMPSTE24 siRNA (Dharmacon, Lafayette, USA) or scramble siRNA was transfected into U2OS cells using HiPerfect transfection reagent (Qiagen, Hilden, Germany).

### Antibodies

Primary antibodies were sourced as follows: α-Tubulin (ab18251), GFP (ab6673), Nucleophosmin (ab10530), PML (ab179466), γH2AX (ab26350), DIAPH3 (ab245660), pKAP1 (S824) (ab70369) (Abcam, Cambridge, UK); γH2AX (2577) (Cell Signalling Technology); β-actin C4 (SC-47778) (Santa Cruz Biotechnology, Dallas, USA); FLAG (M2, F3165) (Sigma).

### Immunofluorescence and microscopy

U2OS cells were cultured on 1.5H glass coverslips and fixed in 4% paraformaldehyde in PBS for 10 min at 21 °C followed by 5 min permeabilization with 0.5% NP-40 in PBS or 10 min in 100% methanol at −20 °C. Coverslips were then blocked (3% BSA in PBS) for 1 h at 21 °C before incubation with primary antibodies in a blocking solution for 12 h at 4 °C in a humidifying chamber. Coverslips were washed in PBS followed by 1 h 21 °C incubation with fluorescent dye-conjugated secondary antibodies (Invitrogen, Waltham, USA). Coverslips were washed with PBS, mounted onto slides with medium containing DAPI (4′,6-diamidino-2-phenylindole), and z stacks were obtained using a Nikon A1R confocal microscope with 60x objective using NIS-Elements software. Different channels were acquired sequentially.

### Western blot

Cell extracts were harvested by washing U2OS cells in ice-cold PBS, then scraping them into fresh ice-cold PBS and centrifugation at 1000×*g* for 5 min. Pellets were resuspended in lysate buffer (10 mM Tris pH 7.5, 150 mM NaCl, 1 mM EDTA, 1% Triton X-100, protease inhibitors) and sonicated for 10 s followed by centrifugation at 1000×*g* for 5 min. 0.01% bromophenol blue, 200 mM DTT, 4% SDS and 20% glycerol were added to lysates before boiling for 5 min, separation by SDS-PAGE and transfer to PVDF (polyvinylidene fluoride) membrane. Membranes were blocked with 5% milk or BSA in PBS followed by primary antibody incubation for 16 h at 4 °C. Membranes were washed with TBST prior to 1 h incubation at 21 °C with secondary antibodies conjugated to IRDye 800CW (LI-COR Biosciences, Lincoln, USA) and final TBST/TBS washes. Odyssey Imager (LI-COR) was used for detection and quantification.

### Nuclear and biochemical cell fractionations

Nuclear fractionation: U2OS cells were incubated in fractionation buffer A (10 mM HEPES pH 7.9, 10 mM KCl, 1.5 mM MgCl_2_, 0.5 mM DTT, 0.05% NP-40, protease inhibitors and phosphatase inhibitors) on ice for 30 min with occasional agitation. The lysate was then passed through a 27 gauge needle ten times, followed by centrifugation at 1500×*g* for 5 min at 4 °C. The supernatant was collected (cytoplasmic fraction) and the remaining pellet was resuspended in IP buffer (10 mM Tris pH 7.5, 150 mM NaCl, 1 mM EDTA, 1% Triton X-100, protease inhibitors) and sonicated for 10 s on ice. Samples were then centrifuged at 3000×*g* for 5 min at 4 °C and supernatant (nuclear fraction) recovered.

Biochemical cell fractionation: Approximately 2 × 10^6^ U2OS cells were washed with ice-cold PBS, then resuspended in fractionation buffer A and incubated on ice for 30 min with agitation. Samples were centrifuged at 1500×*g* for 5 min at 4 °C and supernatant (cytoplasmic fraction) was collected. Pellets were resuspended in fractionation buffer B (50 mM HEPES pH 7.5, 150 mM NaCl, 1 mM EDTA, 10 mM NaF, 10 mM β-glycerophosphate, 1 mM sodium orthovanadate, protease inhibitors) and incubated for 30 min at 21 °C with occasional agitation. Samples were centrifuged as before and the supernatant (nuclear soluble fraction) was collected. Pellets were then resuspended in fractionation buffer C (50 mM HEPES pH 7.5, 150 mM NaCl, 1 mM EDTA, 10 mM NaF, 10 mM β-glycerophosphate, 1 mM sodium orthovanadate, 300 µg/ml DNase I, protease inhibitors) for 30 min at 37 °C and centrifuged as before. The supernatant was collected (chromatin-associated fraction) and pellets were resuspended in IP buffer and sonicated for 10 s on ice, followed by final centrifugation and collection of supernatant (nuclear envelope/insoluble fraction).

### DNA damage time-course assay

U2OS cells that had been induced to express either EGFP or EXP6 by adenovirus were pre-sensitised with 10 µM BrdU for 24 h and then irradiated with 50 J/m^2^ UV using a UV Stratalinker^®^ 1800 (Stratagene^®^, California, USA). Cells were harvested at various time points between 0–36 h and levels of DNA damage were assessed by quantification of γH2AX.

### Cell vitality assays

Cell vitality assays were performed using Vitality (VB-48) protocol on a Nucleocounter NC-3000 reader (ChemoMetec, Denmark). Experiments were performed according to the manufacturer’s instructions.

### FLAG-IP assays

U2OS cells were transduced with Prelamin A or EGFP (control) adenoviruses and whole cell lysates were obtained by sonicating cells in IP buffer for 10 s on ice and collecting supernatant after centrifugation at 1500×*g* for 5 min at 4 °C. ANTI-FLAG® M2 Affinity Gel slurry (Sigma) was added to 500 µg of lysates and IP buffer was added to a final volume of 1 ml. Samples were incubated at 4 °C, rotating for 2 h. Bead-protein complexes were washed 3x in IP buffer, and following the final wash, the pellet was resuspended in 0.01% bromophenol blue, 200 mM DTT, 4% SDS and 20% glycerol and heated at 100 °C for 10 min before centrifugation and western blot analysis of supernatant.

### GFP-TRAP assays

U2OS cells at 70% confluency were transfected with GFP-nAC or GFP as described. After 24 h cells were washed with ice-cold PBS and then nuclear fractionations were performed as described and 20 µl volumes of nuclear fractions were removed and analysed as ‘input’. GFP-Trap affinity resin (ChromoTek) was washed in IP, then added to remaining nuclear fraction lysates and incubated on a rotating wheel at 4 °C for 2 h. Beads were then washed 3x with IP buffer and treated with Benzonase for 30 min at 21 °C. Samples were centrifuged and resuspended in 0.01% bromophenol blue, 200 mM DTT, 4% SDS and 20% glycerol and heated at 100 °C for 10 min before centrifugation and collection of supernatant that was analysed by western blot.

### Statistics

Results are presented as mean values ± SEM. Statistical analysis was performed with GraphPad software. All data were tested for normalcy using the Shapiro–Wilk test before comparison analysis. For comparisons of multiple groups, one-way ANOVA with the Tukey test was used. For the comparison of just two independent samples, the parametric Student *t*-test was used. On graphs, *P < 0.05, **P < 0.01, ***P < 0.001 and ****P < 0.0001. Results were taken from a minimum of three independent experiments.

## Results

### DNA damage causes the formation of nuclear actin filaments that associate with γH2AX and are required for an efficient DDR

**Fig. 1:**
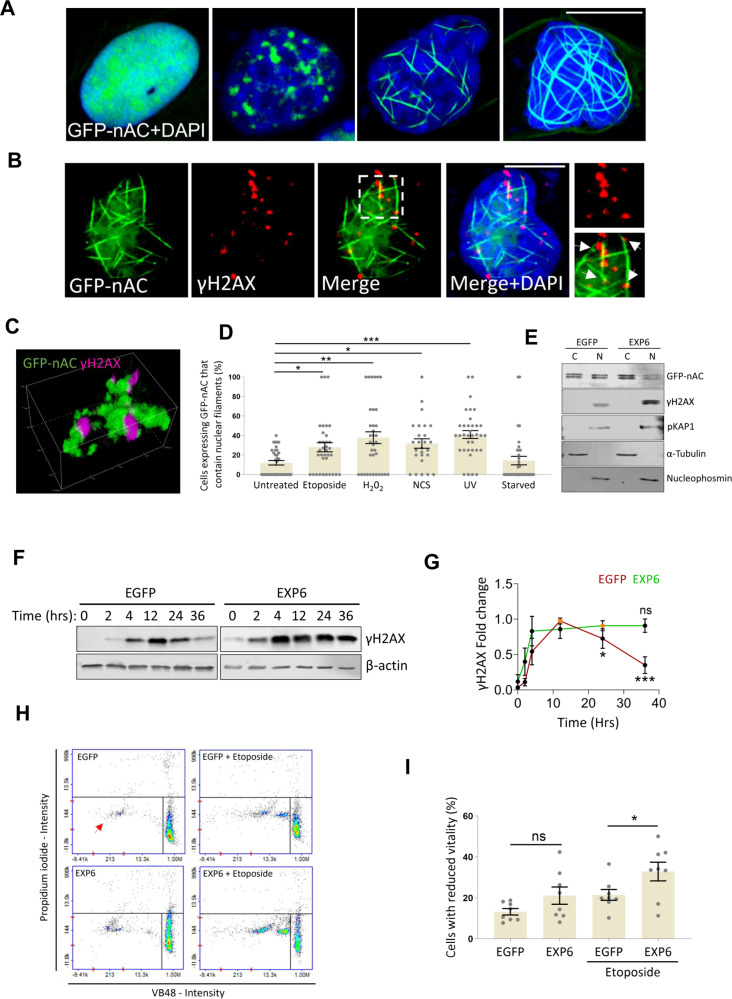
DNA damage causes the formation of nuclear actin filaments that associate with γH2AX and are required for an efficient DDR. **A** Immunofluorescence (IF) showing nuclear actin structures (green) was observed in U2OS cells expressing GFP-nAC (U2OS + GFP-nAC). DNA is stained with DAPI (blue), scale bar is 10 µm. **B** IF showing the association between γH2AX (red) and nuclear F-actin (green) in U2OS cells expressing GFP-nAC and treated with 1 mM etoposide for 3 h (colocalisation indicated by white arrows). DNA is stained with DAPI (blue), scale bar is 10 µm. **C** 3D image is taken from confocal IF analysis of nuclear F-actin (green) and γH2AX (magenta) in a U2OS cell expressing GFP-nAC that were fixed 1 h post irradiation with 50 J/m^2^ UV. **D** Quantification of IF comparing nuclear F-actin accrual in U2OS + GFP-nAC cells treated with etoposide (1 mM for 3 h), hydrogen peroxide (H_2_O_2_) (200 µM for 2 h, Neocarzinostatin (NCS) (0.8 µg / ml for 2 h), ultra-violet (UV) irradiation (fixed 1 h after 50 J/m^2^ irradiation) or serum-starved. Data were taken from three independent experiments (*n* > 100 cells). **E** Representative Western blot (WB) from a nuclear fractionation of U2OS + GFP-nAC cells expressing EGFP (control) or mCherry-EXP6. C cytoplasmic fraction and N nuclear fraction. **F** Example WB from DNA damage time-course assay. U2OS cells expressing either EGFP (control) or EXP6 were treated with 10 µM BrdU for 24 h and then irradiated with 50 J/m^2^ UV. Cells were harvested at various time points and γH2AX was quantified. β-actin was used as a loading control. **G** Quantification of DNA damage time-course assay showing fold change of γH2AX normalised to the highest intensity for each treatment (orange data points). **H** Cell vitality assay comparing cell health of U2OS cells expressing EGFP (controls) or mCherry-EXP6 (EXP6) and treated with or without 1 mM etoposide for 3 h. Healthy cells are present in the bottom right quadrant, the red arrow shows cells with reduced vitality. **I** Quantification of cell vitality assays shown in **H** (*n* = 8). Data were presented as mean ± SEM and were analysed by one-way ANOVA and Tukey’s test. *p < 0.05, **p < 0.01 and ***p < 0.001. ns not significantly different.

Recent technological advances have allowed the detection and visualisation of nuclear actin. We utilised a chromobody consisting of an actin V_HH_ domain, GFP-tag and nuclear localisation sequence (GFP-nAC) to directly visualise nuclear actin in U2OS cells. We observed multiple variations of F-actin structures in ~20% of transfected cells, ranging from short punctate aggregates to large convoluted meshworks that together may represent different stages of F-actin polymerisation (Fig. 1A).

Nuclear F-actin is involved in DNA repair and has been shown to interact with DDR factors [42]. However, there is little evidence to show filaments associated with sites of DNA repair. Immunofluorescence (IF) of U2OS cells expressing GFP-nAC (U2OS + GFP-nAC) and treated with etoposide showed a clear association between nuclear F-actin and γH2AX, with the later ‘budding’ off actin filaments and frequently appearing at actin rod termini (Fig. 1B, C).

Polymerisation of nuclear F-actin occurs in response to DNA DSBs [12, 27] and limited evidence shows base alterations can also result in F-actin formation [12]. As DNA repair is multi-faceted and the precise mode of repair—and factors involved in repair—depends on the type of DNA lesion, we wanted to characterise if actin filaments increased following different types of DNA damage in our model. We treated U2OS + GFP-nAC with etoposide (DNA DSBs), H_2_O_2_ or Neocarzinostatin (NCS) (both oxidative DNA damage) or ultra-violet (UV) radiation (DNA bulky adducts) alongside serum starvation, and then enumerated cells presenting with nuclear F-actin to assess if these different stressors influenced nuclear actin polymerisation (Fig. 1D). We observed an increase in nuclear F-actin in cells treated with all three DNA damaging treatments to a similar extent with etoposide inducing a 2.3-fold increase in cells positive for nuclear filaments, H_2_O_2_ a 2.7-fold increase, NCS a 2.4-fold increase and UV a 3.4-fold increase; indicating filamentous nuclear actin is involved in multiple DNA repair pathways. We did not detect changes in nuclear F-actin following serum starvation, suggesting not all types of cell stress affect nuclear F-actin.

Should nuclear F-actin be a critical component of DNA repair, we anticipated that loss of functionality would reduce DDR efficiency and give rise to unrepaired DNA lesions. In order to disrupt nuclear actin whilst not affecting cytoplasmic actin, we used adenovirus to over-express Exportin 6 (EXP6) [6] in U2OS + GFP-nAC cells to deplete nuclear actin pools (Figs.S1A, B). We then quantified levels of DNA damage in these cells by measuring levels of γH2AX and found loss of nuclear actin resulted in higher levels of DNA damage (Fig. 1E and S1C, D), suggesting the DDR had been compromised. To understand how the loss of nuclear actin affected DNA repair dynamics, a time-course experiment was employed that compared the formation and resolution of γH2AX between control U2OS cells and U2OS cells over-expressing EXP6 (Fig. 1F, G). We observed a significant reduction in the ability of EXP6-expressing cells to resolve γH2AX, showing that DNA damage repair efficiency was reduced in these cells, and thereby indicating a loss of nuclear actin compromises the repair of DNA lesions. To assess how depleted nuclear actin affected cell health, we performed cell vitality assays that showed cells over-expressing EXP6 had reduced cell vitality when treated with etoposide (Fig. 1H, I), an indication that loss of nuclear actin makes cells more sensitive to DNA damage stressors.

### PML NBs localise along nuclear actin filaments

**Fig. 2:**
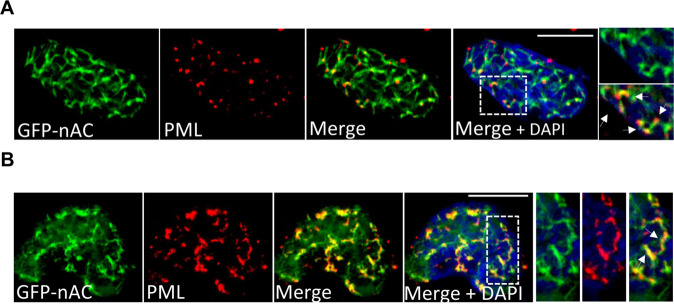
PML NBs localise along nuclear actin filaments. **A** Representative IF image of a U2OS + GFP-nAC cell showing actin (green) co-stained with PML (red). Colocalisation is evident by yellow staining (white arrows). **B** Additional example of nuclear actin and PML colocalization. The spreading of PML along F-actin is visible (white arrows). DNA is stained with DAPI (blue). Scale bars = 10 µm.

Despite the involvement of nuclear F-actin in the DDR, few repair factors have been found to directly associate with these filaments. Recently, proliferating cell nuclear antigen (PCNA)—a protein involved in the repair of stalled DNA replication forks—was identified to localise to nuclear F-actin and migrate along filaments [43]. This finding led us to examine if other DNA repair proteins might also use this nuclear framework as part of their activity. PML NBs have an undefined role in the DDR but are thought to act as storage hubs for DDR proteins involved in multiple DNA repair pathways [44, 45]. We analysed U2OS + GFP-nAC cells by IF and observed PML formed distinct nuclear bodies that localised along nuclear actin filaments (Fig. 2A and S2A, B). We also found in some cells with PML spread out along nuclear F-actin (Fig. 2B), which made us consider if these filaments acted as a substrate for PML motility. To test this, we co-expressed an mCherry-PML recombinant protein which localised to nuclear F-actin (Fig. S2C), however timelapse microscopy failed to show the clear movement of PML along filaments (Fig. S2D).

### Prelamin A causes mislocalisation of nuclear actin

Prelamin A is a toxic precursor to lamin A that is documented to accumulate at the NE of certain cell types with age [46, 47]. Expression of this immature lamin contributes to genomic instability via dysfunction of the DDR and eventual activation of senescence or apoptosis [48].

**Fig. 3:**
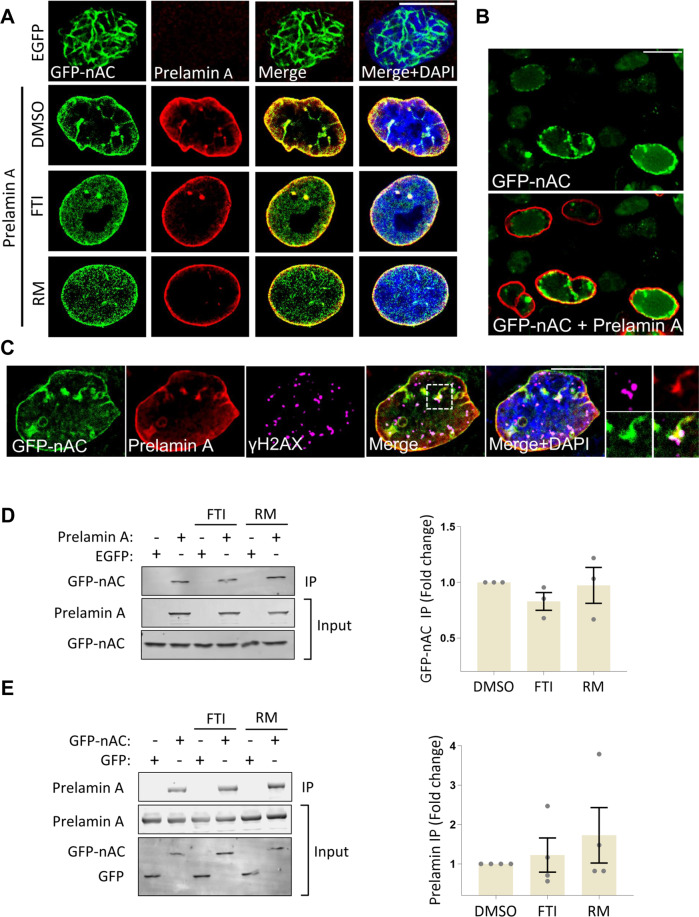
Prelamin A causes mislocalisation of nuclear actin. **A** Representative IF images of U2OS + GFP-nAC cells over-expressing prelamin A or EGFP control. Prelamin A (red) causes mislocalisation of nuclear actin (green) to the nuclear envelope (NE). This is not rescued by either farnesyltransferase inhibitor (FTI) or Remodelin (RM) treatment. **B** IF of U2OS cell line stably expressing GFP-nAC and over-expressing prelamin A (red). The same NE mislocalisation of nuclear actin can be observed. **C** IF showing triple staining of nuclear actin (green), prelamin A (red) and γH2AX (magenta) in U2OS + GFP-nAC cells. γH2AX is observed trapped in nucleoplasmic reticulum (magnified image) and at the NE alongside prelamin A and actin but the majority of foci no longer associate with the nuclear F-actin network. DNA is stained with DAPI (blue). All scale bars = 10 µm. **D** Representative WB and quantification of FLAG-IP assays investigating prelamin A and nuclear actin interactions in U2OS cells expressing FLAG-tagged prelamin A and GFP-nAC and treated with DMSO, FTI or RM. Cells expressing EGFP and GFP-nAC were used as negative controls (*n* = 3). **E** GFP-trap representative WB and quantification of assays using GFP-nAC as bait to test for interactions with prelamin A in U2OS cells expressing GFP-nAC and prelamin A. Cells expressing GFP instead of GFP-nAC were used as negative controls. The effect of adding FTIs and RM on nuclear actin–prelamin A interactions was tested (*n* = 4). Data were presented as mean ± SEM and were analysed by one-way ANOVA and Tukey’s test.

Interestingly, mature lamins [39, 40] and the NE protein emerin [41] interact with nuclear actin, implying the NE might be important for nuclear actin function. We, therefore, wanted to test if disruption of the NE by prelamin A expression affected nuclear F-actin, and if these changes could result in a defective DDR. For this, we used adenoviral transduction to express prelamin A in U2OS + GFP-nAC cells and used IF to assess nuclear actin localisation. Our results showed a striking change in nuclear actin positioning, with a marked shift of actin to the NE and intranuclear invaginations, and almost complete ablation of nuclear filaments (Fig. 3A). Treatment with either farnesyltransferase inhibitor 276 (FTI) [49] or Remodelin (RM) [50], two compounds were previously shown to alleviate some aspects of prelamin A expression, did not improve organisation of nucleoplasmic F-actin despite rescuing nuclear morphology. Importantly, the stress-associated increase in nuclear F-actin seen after DNA damage induction did not occur in cells expressing prelamin A (Fig. S3A). Prelamin A was also expressed in a U2OS cell line stably expressing the nuclear actin chromobody and the same mislocalisation of actin was observed (Fig. 3B). Displacement of nuclear actin by prelamin A was confirmed using biochemical fractionation. (Fig. S3B, C). Further IF using triple staining indicated some γH2AX still co-stained with nuclear actin and more γH2AX foci were present at the NE alongside mislocalised actin, however, the overall association between both had been drastically reduced by prelamin A expression (Fig. 3C and S3D).

Despite containing two nuclear actin-binding domains, in vitro studies have shown prelamin A binds less efficiently to nuclear actin compared to mature lamins [40]. However, as our data showed prelamin A caused nuclear actin translocation to the NE, we hypothesised this could be caused by a direct interaction. To investigate this, FLAG immunoprecipitation assays (Prelamin A as bait) (Fig. 3D) and GFP-TRAP assays (actin as bait) (Fig. 3E) were used in U2OS + GFP-nAC cells expressing Prelamin A and these showed both proteins were in complex with each other. Treatment with either FTI or RM had no influence on this interaction.

### Expression of prelamin A ablates PML localisation on nuclear F-actin

**Fig. 4:**
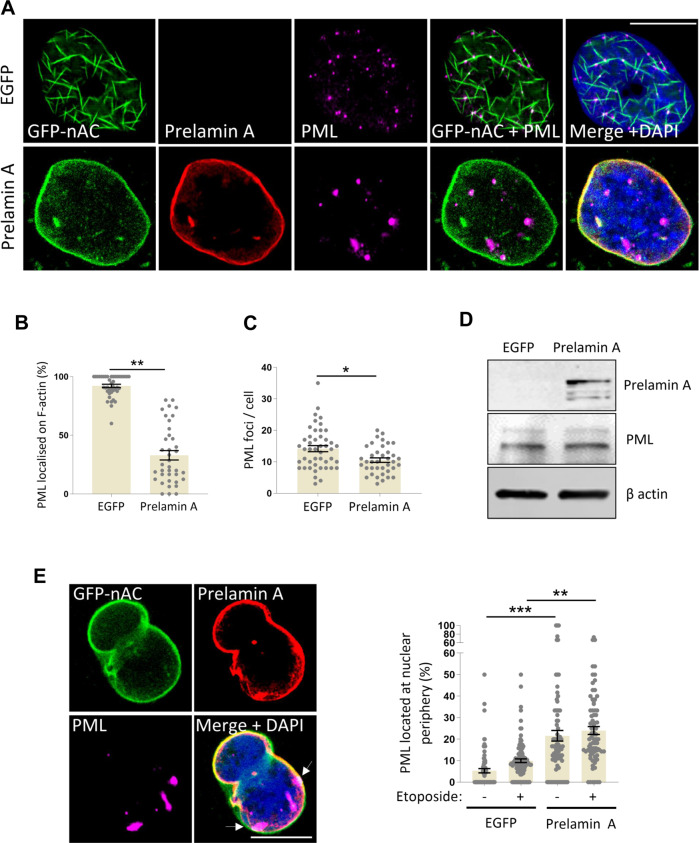
Expression of prelamin A ablates PML localisation on nuclear F-actin. **A** Representative IF images comparing nuclear actin (green) and PML (magenta) colocalisation in U2OS + GFP-nAC with and without prelamin A expression (red). PML-actin interactions are prevented by prelamin A and PML NB foci in these cells are less numerous and appear larger compared to prelamin A negative cells. **B**, **C** Quantification PML localisation and enumeration from IF shown in **A**, *n* = 5 (>100 cells). **D** Representative WB showing the expression of prelamin A does not affect the level of PML in U2OS cells (quantification is shown in Supplement Fig. 4B). **E** IF data showing that prelamin A expression in U2OS + GFP-nAC cells increases the localisation of PML NBs at the nuclear periphery (white arrows) with or without additional DNA damage (1 mM etoposide for 3 h). DNA is stained with DAPI (blue), *n* = 4 (>100 cells). Data were presented as mean ± SEM and were analysed by one-way ANOVA and Tukey’s test. *p < 0.05, **p < 0.01 and ***p < 0.001. Scale bars in IF images = 10 µm.

The stark repositioning and loss of nuclear actin filaments caused by prelamin A led us to test if PML functionality was also affected by this toxic lamin precursor. IF showed that, unlike nuclear actin, PML remained largely in intranuclear aggregates following prelamin A expression (Fig. 4A and S4A) with quantification revealing a significant decrease of PML associated with nuclear F-actin (Fig. 4B). Additionally, PML NB morphology was notably altered with bodies appearing larger and fewer in number (Fig. 4C), despite no change in overall PML protein (Fig. 4D and S4B).

Although PML was still presented as foci throughout the nucleoplasm of prelamin A positive cells, significantly more of these foci were found close to the NE (Fig. 4E). We postulate some of these PML NBs may still be in complex with actin and, therefore will have been repositioned at the NE through the prelamin A mediated relocalisation of nuclear actin.

### Disruption of nuclear actin via prelamin A or exportin 6 expression causes deregulation of PML during the DDR

**Fig. 5:**
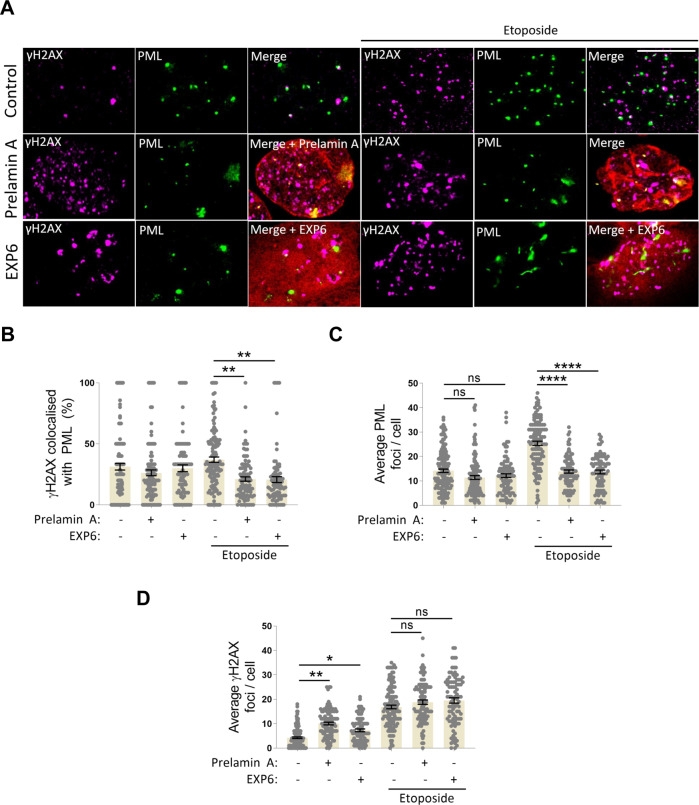
Disruption of nuclear actin via prelamin A or exportin 6 expression causes deregulation of PML during the DDR. **A**–**D** IF and quantification of the effect of over-expressing prelamin A (red) or Exportin 6 (red) on numbers of PML foci (green) and colocalisation of PML NBs with γH2AX (magenta) in U2OS cells treated with or without 3 h treatment with 1 mM etoposide. DNA is stained with DAPI (blue), scale bar = 10 µm. Enumeration of γH2AX for each treatment is also shown. Data were obtained from five independent experiments (>100 cells), are presented as mean ± SEM and were analysed by one-way ANOVA and Tukey’s test. *p < 0.05, **p < 0.01 and ****p < 0.0001. ns not significantly different.

Our observation that prelamin A caused the dissociation of PML NBs from nuclear actin filaments and caused some translocation to the NE led us to examine if PML functionality during the DDR was impeded in these cells. To test this we used triple staining IF to analyse PML fission and localisation to sites of DNA damage in U2OS + GFP-nAC cells expressing prelamin A. We found that in cells treated with etoposide, prelamin A significantly attenuated PML colocalization with γH2AX (Fig. 5A, B) and reduced microbody formation in response to DNA damage (Fig. 5A, C) despite the highest levels of γH2AX being detected in cells expressing prelamin A (Fig. 5A, D). Importantly, the same phenomena were observed following the exportin 6 over-expression (Fig. 5A–D), indicating the changes in PML seen following either the over-expression of prelamin A or exportin 6 were caused by the displacement of the nuclear F-actin network. However, unlike prelamin A, exportin 6 did not cause PML localisation at the NE.

### Leptomycin B restores nuclear F-actin networks in prelamin A-expressing cells and improves genomic integrity when used in combination with Remodelin

We hypothesised that if nuclear F-actin could be restored in prelamin A positive cells, PML function might also improve and therefore be more capable to perform its role in the DDR, leading to restored genome stability. We explored different avenues to achieve this, including over-expression of wild-type lamin A (to compete against prelamin A—nuclear actin interactions), retinoic acid receptor (RAR) agonists and antagonists (as modulation of the RAR pathway has been shown to alleviate adverse effects associated with lamina dysfunction [51, 52]) and also over-expression of the dominant-negative nesprin KASH (Klarsicht, ANC-1, Syne-Homology domain) domain—in order to disrupt mechanotransduction [53], but none of these treatments showed any signs of improving prelamin A induced DDR disruption (Fig.S5A–C).

Polymerisation of nuclear actin in response to stress is dependent on the activity of mDia formins [54, 55]. Thus, the dynamics of nuclear F-actin rely not only on the concentration of actin in the nucleus, but also on polymerisation factors. Formin mDia2 is a critical component that augments F-actin and the nuclear concentration of mDia2 can be increased by preventing its nuclear export via inhibition of exportin 1 using Leptomycin B (LMB) [56]. We examined mDia2 in U2OS cells treated with LMB and found a significant increase in nucleoplasmic mDia2 as anticipated (Fig. S5D).

**Fig. 6:**
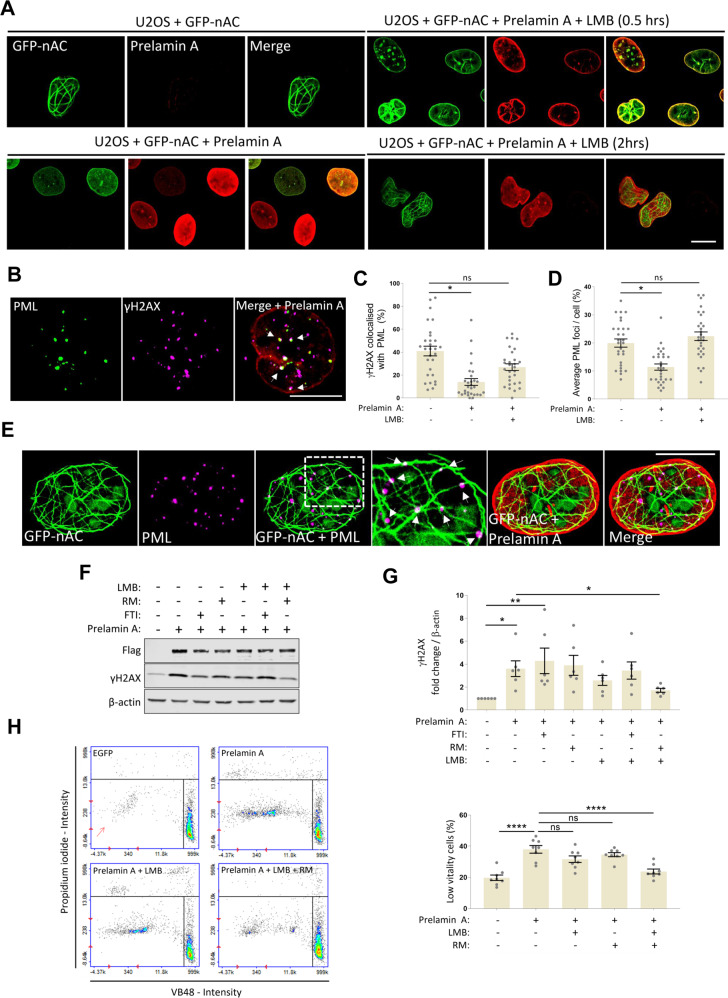
Leptomycin B restores nuclear F-actin networks in prelamin A-expressing cells and improves genomic integrity when used in combination with Remodelin. **A** IF showing the effect of Leptomycin B (LMB) on restoring nuclear F-actin networks in U2OS + GFP-nAC cells. These cells were transduced to express prelamin A (red), causing mislocalisation of nuclear actin (green) to the NE. Treatment with LMB resulted in increased nucleoplasmic nuclear actin structures. **B** IF showing a U2OS cell expressing prelamin A and treated with LMB exhibiting restored colocalisation between PML NBs and γH2AX (white arrows). **C**, **D** Quantification of experiments shown in **B**, both PML*-*γH2AX colocalisation and PML NB foci enumeration were analysed, *n* = 4 (>100 cells). **E** IF showing restoration of PML (magenta) association with nuclear F-actin (green) in a U2OS + GFP-nAC cell also expressing prelamin A (red) that has been treated with LMB. White arrows show PML associating with nuclear F-actin despite prelamin A present at the NE. All scale bars = 10 µm. **F** Representative WB showing levels of DNA damage (γH2AX) in U2OS cells expressing prelamin A that were treated with FTI, RM, LMB or combination treatment. **G** Quantification of the experiment shown in **F**, *n* = 5. **H** Cell vitality data showing the effect of co-treatment of LMB and RM on improving the health of U2OS expressing prelamin A, *n* = 8. Data were presented as mean ± SEM and were analysed by one-way ANOVA and Tukey’s test. *p < 0.05, **p < 0.01 and ****p < 0.0001. ns not significantly different.

We wanted to test if the nuclear accumulation of mDia2 via LMB treatment could promote F-actin formation in cells expressing prelamin A. Nuclear actin in U2OS + GFP-nAC cells that had been transduced to express prelamin A and further treated with LMB was assessed by IF and a significant increase in nuclear actin filaments was observed after 2 h treatment with LMB (Fig. 6A and S5E). Interestingly, these cells still presented with actin staining at the NE, suggesting actin interactions with prelamin A remained intact but additional polymerisation of existing pools of actin caused by accumulated mDia2 allowed more filaments that converged into meshworks similar to those seen in controls.

We next determined if the deregulation of PML in response to DNA damage had been mitigated by the rescue of nuclear F-actin networks by LMB. We treated the same cells with etoposide and probed for both PML and γH2AX and found PML recruitment to γH2AX and PML foci numbers had been restored in prelamin A positive cells by LMB (Fig. 6B–D). It was also evident that PML localisation to nuclear actin filaments had vastly improved following LMB treatment (Fig. 6E and S5F, G). To test if this resulted in a more efficient DDR, we employed WB to measure γH2AX in U2OS cells expressing prelamin A that had been treated with either LMB, FTI or RM (Fig. 6F, G). Prelamin A expression caused a significant increase in γH2AX, which was not reduced by any agents alone, however, when LMB was used in combination with RM, levels of γH2AX were significantly lower. Finally, to assess if treating cells with LMB alongside RM had a beneficial effect on cell health, we utilised cell vitality assays and again found that individually neither agent was able to restore cell health, but together they showed a significant improvement in cell vitality (Fig. 6H and S5H). Taken together, these data show that restoration of nuclear F-actin networks by LMB in cells expressing prelamin A improves PML function in the DDR but additional treatment with RM is required to decrease levels of DNA damage to levels comparable to control cells.

## Discussion

### Nuclear actin responds to a myriad of DNA damage lesions and associates with yH2AX

To date, a defined role for nuclear F-actin in DNA repair has only been outlined for the repair of DSBs; however, our data—and previous findings [12]—show that nuclear actin polymerises in response to various types of DNA damage and is thus probably involved in resolving multiple types of DNA lesions. This is further supported by our observation that actin filaments associate with γH2AX caused by genotoxic agents that don’t produce DNA double-strand termini (Fig. 1C). One potential function that nuclear actin filaments could provide in response to DNA damage would be to act as transport networks to facilitate rapid recruitment of repair factors to sites of damaged DNA. Currently, there is little data to support this, however, the DNA replication factor PCNA has been shown to move along nuclear actin filaments [43] and therefore shows these networks can be utilised for translocation.

We identified that PML NBs localised along nuclear F-actin filaments and that nuclear actin was important for PML fission and repositioning at DNA lesions following genotoxic insult. It was also evident that PML could spread along actin filaments, hinting that these structures might be used for motility, however, attempts to detect PML movement along actin filaments using timelapse microscopy proved unsuccessful. Whilst it remains undefined just how nuclear F-actin regulates PML in response to DNA damage, our data conclusively shows that its disruption—either through depletion of nuclear actin or over-expression of prelamin A—causes deregulation of PML.

### Prelamin A causes F-actin mislocalisation and PML dysfunction

Prelamin A inflicts a myriad of harmful effects upon a cell, including nuclear transport defects [36], alterations to chromatin organisation [57] and genomic instability [37]. Our results show that in addition to these changes, prelamin A also disrupts nuclear actin function by inducing actin accumulation at the NE. As a consequence, in response to DNA damage induction, nucleoplasmic F-actin filament networks are unable to form or contribute to DNA repair processes.

The aggregation of nuclear actin at the NE is likely to be via the direct binding of actin to conserved sites within the Ig-fold domain of prelamin A [40]. We propose that the interactions between nuclear actin and the more soluble mature lamin A protein are out-competed by prelamin A once a specific threshold of prelamin A has been reached. As prelamin A is tethered to the NE via its hydrophobic farnesyl group, this heavily restricts the ability of actin to distribute throughout the nucleus even if polymerisation can still occur. The inability of cells to develop nuclear F-actin networks following prelamin A expression may contribute to genomic instability by compromising DNA repair, either through reduced chromatin dynamics or disruption of DNA repair factor activity, such as we have shown with PML.

Previous studies have also shown that disruption of the nuclear lamina can affect PML. Mutations in *LMNA* reportedly lead to fewer PML nuclear bodies in fibroblasts [58]. Additionally, depletion of mature lamins A/C or the lamina-associated protein nesprin-2 was shown to disrupt PML and ERK compartmentalisation in vascular smooth muscle cells, resulting in loss of ATM at DNA lesions [59]. This study also found that prelamin A accumulated at PML NBs following DNA damage. Importantly, PML NB deregulation was also observed in a study investigating the effect of progerin expression [60]. Progerin is associated with Hutchinson Gilford Progeria Syndrome (HGPS) and, like prelamin A is a permanently farnesylated lamin A precursor, but unlike prelamin A, it has a 50 amino acid deletion [61]. Authors identified that PML presented in larger thread-like structures in HGPS cells and that PML NB association with γH2AX was also reduced. Additionally, expression of progerin induced increased localisation of PML NBs to the NE—observations that are strikingly similar to our findings when either prelamin A was expressed or nuclear actin disrupted. We hypothesise that the findings reported in these closely related studies are likely to have been caused by changes in nuclear actin organisation that occurred as a result of the altered nuclear lamina and, as such, underpins the close relationship that the lamina and nuclear actin play in nuclear processes such as DNA repair.

### LMB aids restoration of F-actin networks and reduces genomic instability

We attempted several different approaches to restore stress-induced nuclear F-actin networks in cells expressing prelamin A, including over-expression of mature lamin A, manipulation of retinoic acid signalling and disruption of mechanotransduction by expression of a dominant-negative KASH domain—none of which reduced the cytotoxicity of prelamin A.

We then progressed to test if we could promote nuclear F-actin assembly by increasing the concentration of nuclear formin mDia2 using LMB and found significantly more nuclear F-actin was present that wasn’t localised to the NE. Further analysis of these cells showed PML localisation to filaments was again observed alongside restored PML dynamics following DNA damage. Although LMB treatment alone only showed modest improvements in levels of prelamin A induced DNA damage and cell vitality, when used in combination with the NAT10 acetyl-transferase inhibitor Remodelin—a molecule previously found to counteract cytotoxic properties of non-mature lamins [36, 50]—both these parameters were significantly increased. We propose that the beneficial effects of LMB result from increased polymerisation of existing pools of nucleoplasmic G-actin that do not become anchored at the NE due to saturation of prelamin A already complexed with actin. Formins reside in an autoinhibited state and require activation in order to polymerise actin. As our findings show that the formation of actin nucleoplasmic filaments occurs following the prevention of mDia2 nuclear export, this would mean that the activation of mDia2 is not impeded by prelamin A, nor a lack of actin, but instead on the availability of nuclear mDia2 protein [62].

In this study, we have demonstrated the importance of nuclear F-actin networks on PML NB activity during DNA repair, and how abrogation of these networks by prelamin A causes PML NB dysfunction that contributes to genomic instability. Augmentation of nuclear actin polymerisation by preventing mDia2 nuclear export improved nuclear F-actin formation after stress and restored PML NB function in prelamin A positive cells. Thus, in nuclear actin, we provide a novel target for therapeutic intervention in cells disrupted by non-mature lamins such as in progeria disorders and physiological ageing.

## Supplementary information

Supplement Figure Legends

Supplement Fig 1

Supplement Fig 2

Supplement Fig 3

Supplement Fig 4

Supplement Fig 5

Nuclear F-actin (green) and PML (red) in a U2OS cell

Original Data File

Checklist

Author contributions as a separate file (this is also included in the main text file)

## Data Availability

All datasets generated and analysed during this study are included in this published article and its Supplementary Information files. Additional data are available from the corresponding author on reasonable request.
