# Supplementary material for: Filamentous nuclear actin regulation of PML NBs during the DNA damage response is deregulated by prelamin A
Source: Cell Death Dis. 2022 Dec 15;13(12):1042. doi: 10.1038/s41419-022-05491-4 (PMC9755150; doi:10.1038/s41419-022-05491-4)
Supplement: Supplementary file 1 — Supplement Figure Legends [file 41419_2022_5491_MOESM1_ESM.docx]

**Figure Legends - Supplement**

**Fig.S1 DNA damage causes formation of nuclear actin filaments that associate with γH2AX and are required for an efficient DDR** (**A**) Representative IF image showing over-expression of mCherry-Exportin 6 (red) in U2OS+GFP-nAC. Nuclear actin (green) is exported into the cytoplasm (white arrows). DNA is stained with DAPI (blue). (**B**, **C and D**) Quantification of WB shown in Fig. 1E, showing expression of exportin 6 reduces nuclear actin (**B**) and simultaneously increases levels of DNA damage ($\gamma$H2AX) (**C**) and pKAP1 (**D**), n = 3. Data are presented as mean ± SEM and were analysed by parametric Student *t* test. ** = *p* < 0.01 and *** *p* < 0.001.

**Fig.S2 PML NBs localise along nuclear actin filaments** (**A**) Line-scan plot of IF data shown in Fig.2A showing extent of PML (red) and nuclear actin (green) colocalisation. Graph (right) shows colocalised regions as blue peaks. (**B**) 3D confocal microscopy image of PML in U2OS+GFP-nAC. PML foci (red) are distributed along nuclear F-actin (green). (**C**) IF representative image of recombinant mCherry-PML and nuclear actin (green) in U2OS expressing GFP-nAC. Localisation of PML along nuclear F-actin is evident (white arrows). DNA is stained with DAPI (blue). Scale bar = 10 µm. (**D**) Timelapse of PML (red) and nuclear actin (green) in U2OS expressing GFP-nAC. Images were taken every 2 minutes for 1.5 hours then frameshift adjusted.

**Fig.S3 Prelamin A causes mislocalisation of nuclear actin** (**A**) Quantification of IF data investigating the effect of prelamin A expression on nuclear F-actin polymerisation in U2OS+GFP-nAC treated with various inducers of DNA damage (etoposide (1 mM for 3 hrs), hydrogen peroxide (H_2_O_2_) (200 µM for 2 hours, ultra-violet (UV) irradiation (fixed 1 hour after 50 J/m^2^ irradiation) or serum starved. Data taken from 3 independent experiments (> 100 cells). (**B-C**) Representative WB and quantification of biochemical cell fractionations investigating the effect of prelamin A on nuclear actin cellular localisation. Cy = cytoplasmic fraction, Ns = nuclear soluble fraction, Ch = chromatin fraction, Ne = nuclear envelope fraction. U2OS+GFP-nAC were also transduced to express prelamin A. Prelamin A caused nuclear actin to shift from chromatin fraction into the nuclear envelope fraction as indicated by probing with GFP (actin chromobody). Quantification of this shift is shown in (**C**). (**D**) Quantification of experiments shown in Fig. 3C, showing prelamin A expression reduces localisation of γH2AX on nuclear F-actin in U2OS+GFP-nAC cells in the presence or absence of DNA double-strand breaks caused by 3 hr 1 mM etoposide treatment (n = 4). Data are presented as mean ± SEM and were analysed by one-way ANOVA and Tukey’s test or parametric Student *t* test. * = *p* < 0.05, ** = *p* < 0.01 and *** *p* < 0.001. ns = not significantly different.

**Fig.S4 Expression of prelamin A ablates PML localisation on nuclear F-actin** (**A**) Representative IF showing PML in a U2OS+GFP-nAC cell also expressing prelamin A. Prelamin A (red) localises to the NE and causes nuclear actin (green) to also localise at the nuclear periphery. PML (magenta) no longer associates with nuclear F-actin. DNA is stained with DAPI (blue), scale bar is 10 µm. (**B**) Quantification of WB shown in Fig. 4D showing no change in either PML isoform upon prelamin A expression (adPLA) in U2OS cells, n = 4. Data are presented as mean ± SEM and were analysed by parametric Student *t* test.

**Fig.S5 Leptomycin B restores nuclear F-actin networks in prelamin A expressing cells and improves genomic integrity when used in combination with Remodelin** (**A**) WB analysis of whole cell lysates from U2OS cells expressing prelamin A through siRNA directed depletion of Face1 (siFace1) (causing prelamin A to accumulate). These cells were further treated with adenovirus to over-express wild-type lamin A (WTLA) to examine if mature lamin A could out-compete prelamin A to reduce levels of DNA damage ($\gamma$H2AX), however no improvement was seen, n = 3. (**B**) WB analysis of lysates from U2OS cells expressing prelamin A that were treated with retinoic acid receptor agonist AC261066 (AC) and antagonist BMS453 (BMS) and further treated with etoposide. Levels of DNA damage were assessed by quantifying $\gamma$H2AX, n = 3. (**C**) WB and IF analysis examining the over-expression of the dominant negative nesprin KASH (Klarsicht, ANC-1, Syne-Homology domain) domain in U2OS cells over-expressing prelamin A. There was no decrease in $\gamma$H2AX detected either in WB or IF (magenta staining), suggesting disruption of mechanotransduction was insufficient to restore genomic instability induced by prelamin A. (**D**) Representative IF and quantification showing LMB treatment increases levels of nucleoplasmic mDia2 (green). Data was taken from 3 independent experiments and mDia2 intensity was measured in > 100 cells. (**E**) Quantification of results shown in Fig. 6A. 2 hours of LMB treatment resulted in a significant increase in nuclear F-actin in cells treated with etoposide (1 mM for 3 hrs), n = 3 (> 100 cells). (**F**) Line-scan plot of IF data shown in Fig.6E showing extent of PML and nuclear actin colocalisation. Graph (right) shows colocalisation of PML (magenta) and nuclear actin (green) as blue peaks. (**G**) Additional example IF showing restoration of PML (magenta) association with nuclear F-actin (green) in a U2OS+GFP-nAC cell expressing prelamin A (red) that has been treated with LMB. Scale bars = 10 µm. (**H**) Cell vitality data showing effect of Remodelin treatment on U2OS cells expressing prelamin A and quantification of ‘dead cells’ (indicated by red arrow) from experiment shown in Fig. 6H, n = 8. Data are presented as mean ± SEM and were analysed by one-way ANOVA and Tukey’s test. * = *p* < 0.05, ** = *p* < 0.01 and **** *p* < 0.0001. ns = not significantly different.
