# Supplementary figures and images for: Filamentous nuclear actin regulation of PML NBs during the DNA damage response is deregulated by prelamin A
Source: Cell Death Dis. 2022 Dec 15;13(12):1042. doi: 10.1038/s41419-022-05491-4 (PMC9755150; doi:10.1038/s41419-022-05491-4)

## Slide 1
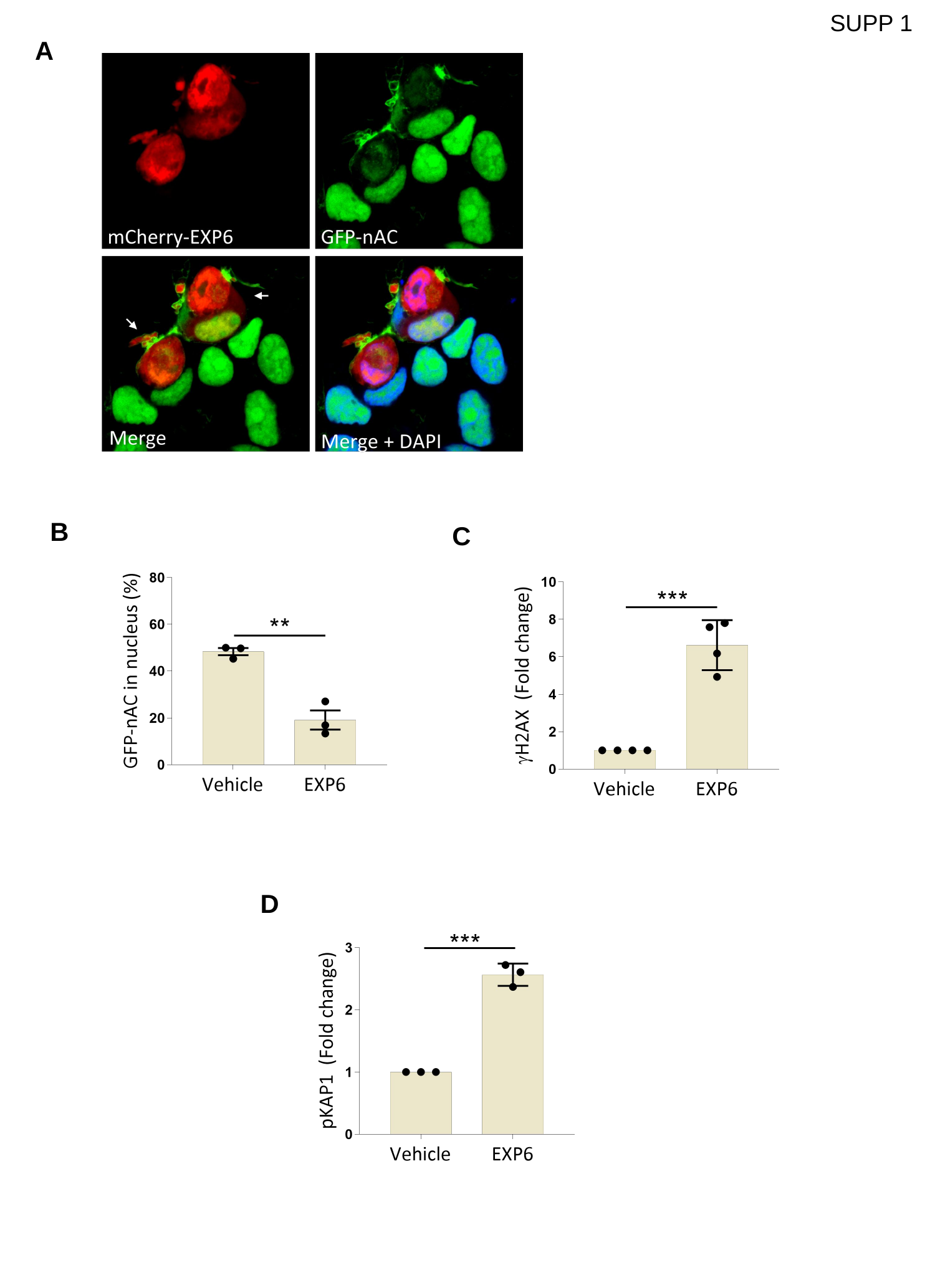

SUPP 1
A
B
C
D

Supplement: Supplementary file 2 — Supplement Fig 1 [file 41419_2022_5491_MOESM2_ESM.ppt]

## Slide 1
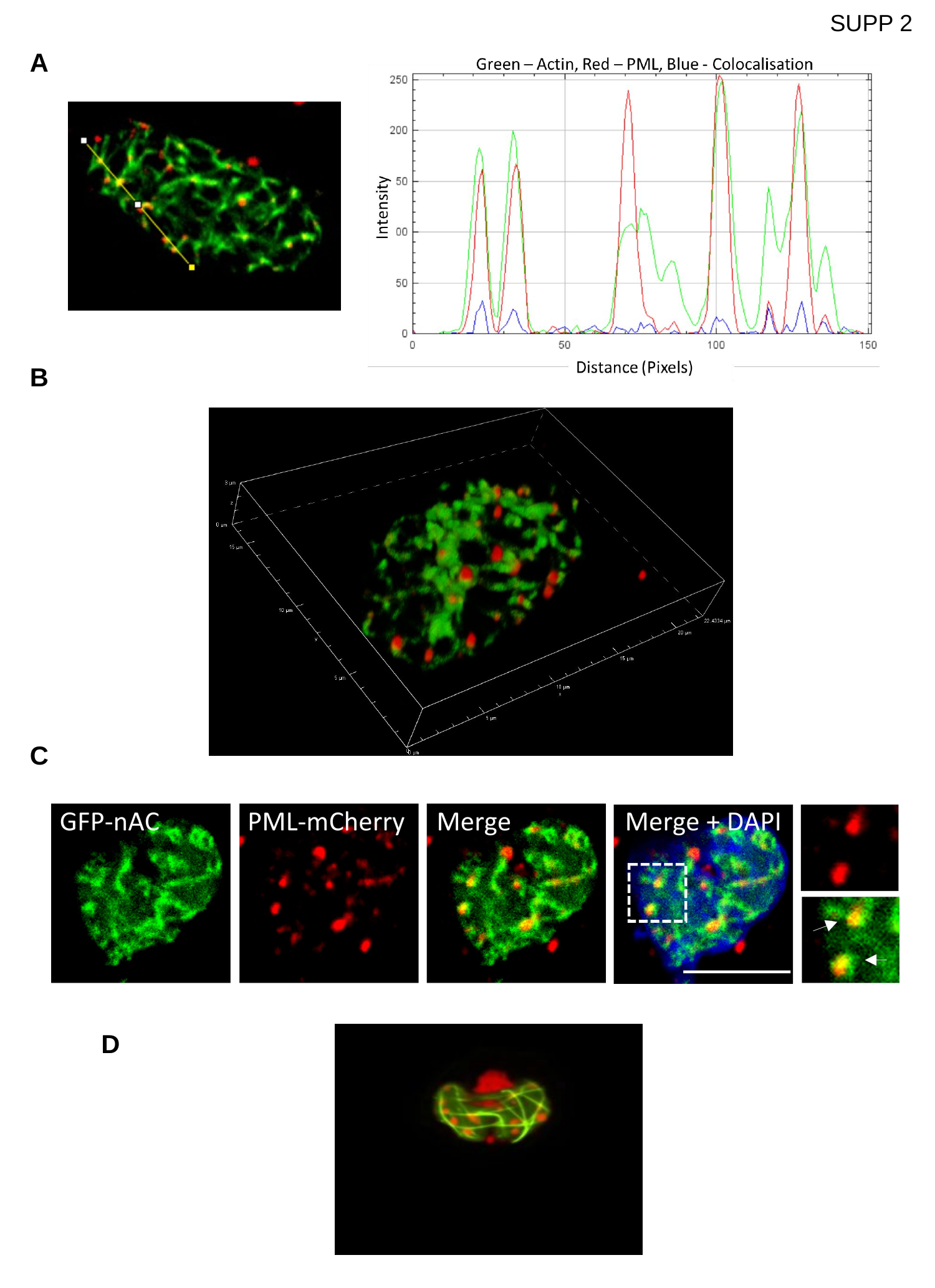

SUPP 2
A
B
C
D

Supplement: Supplementary file 3 — Supplement Fig 2 [file 41419_2022_5491_MOESM3_ESM.ppt]

## Slide 1
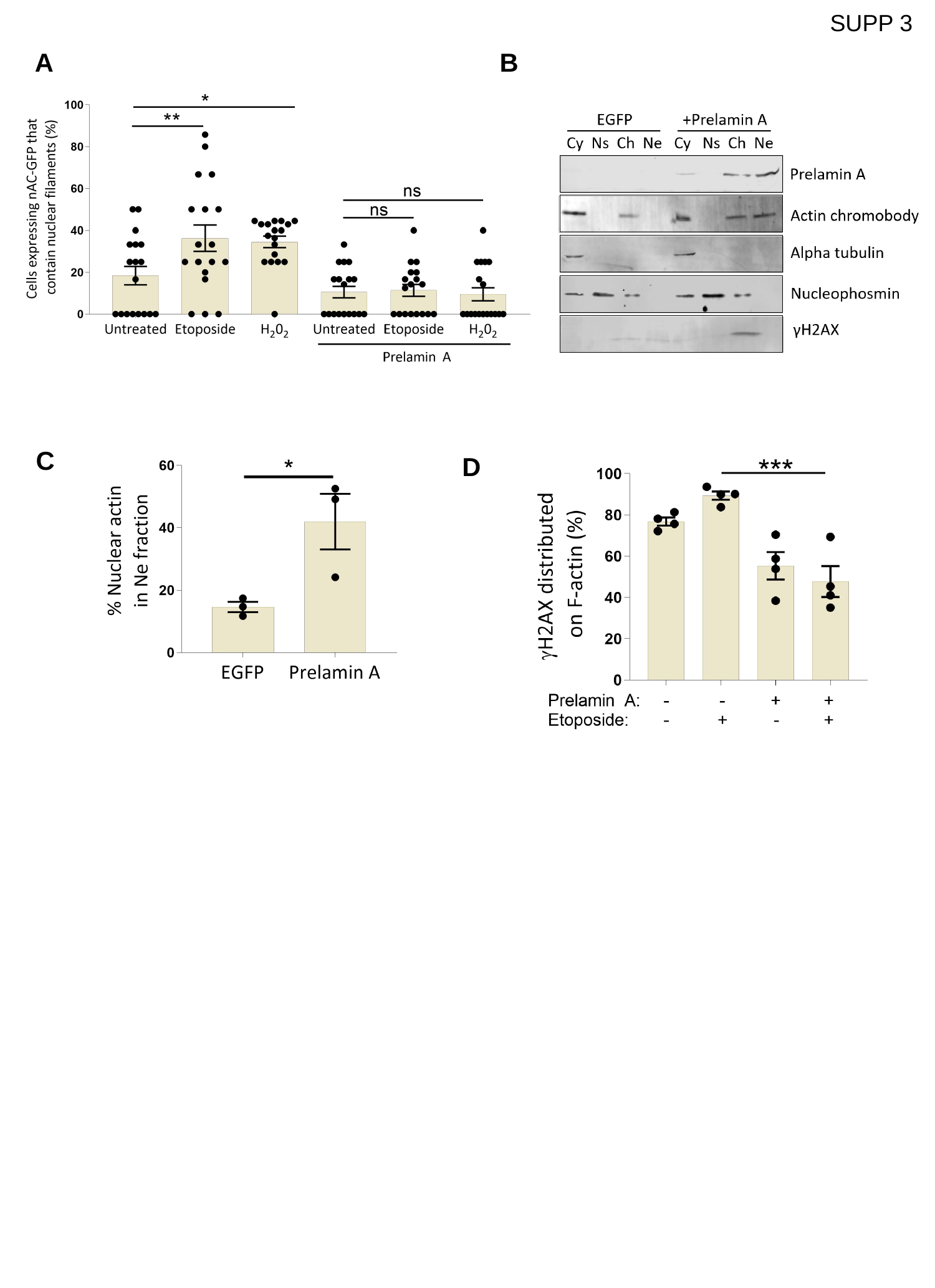

SUPP 3
A
B
C
D

Supplement: Supplementary file 4 — Supplement Fig 3 [file 41419_2022_5491_MOESM4_ESM.ppt]

## Slide 1
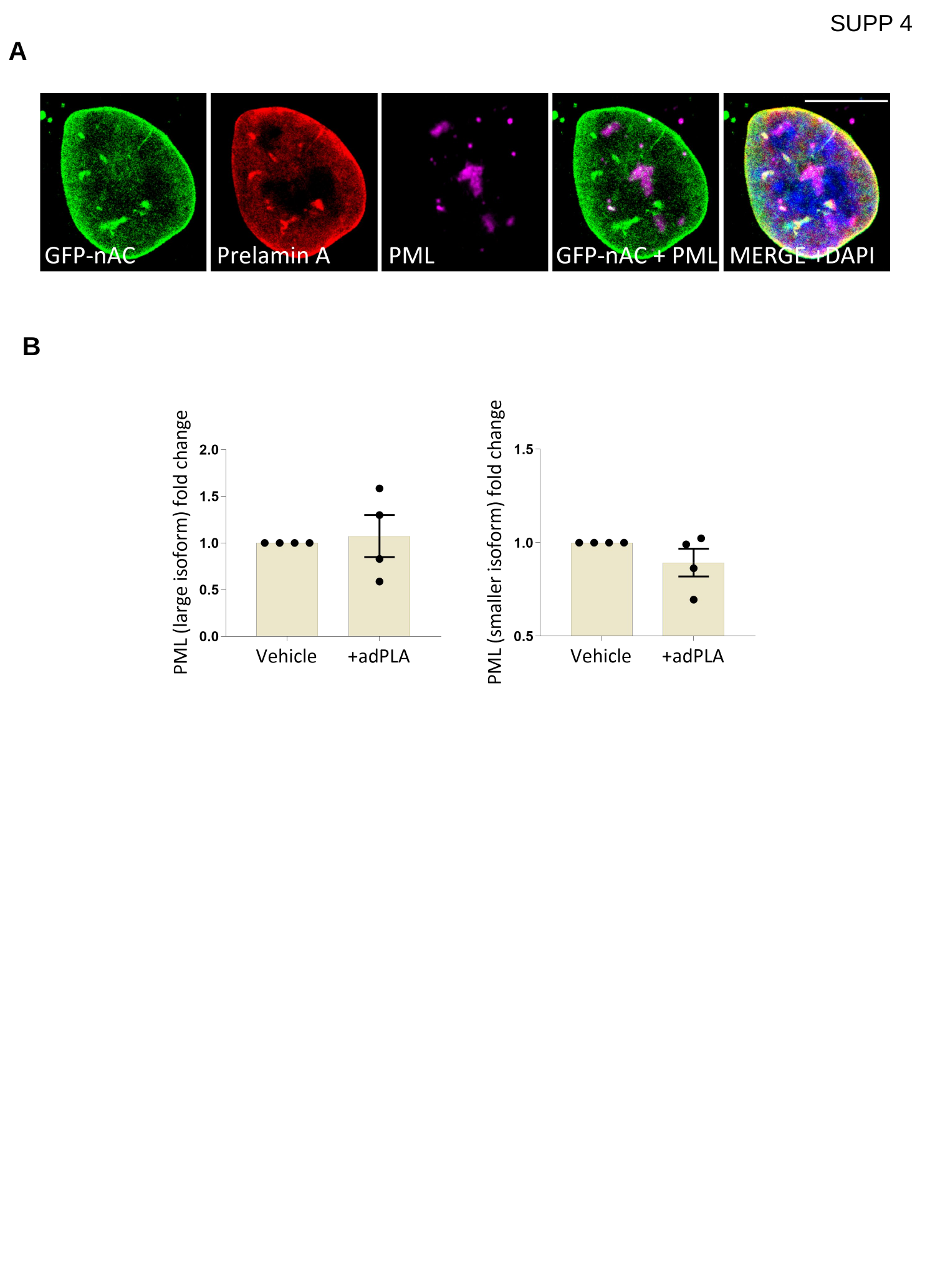

SUPP 4
A
B

Supplement: Supplementary file 5 — Supplement Fig 4 [file 41419_2022_5491_MOESM5_ESM.ppt]

## Slide 1
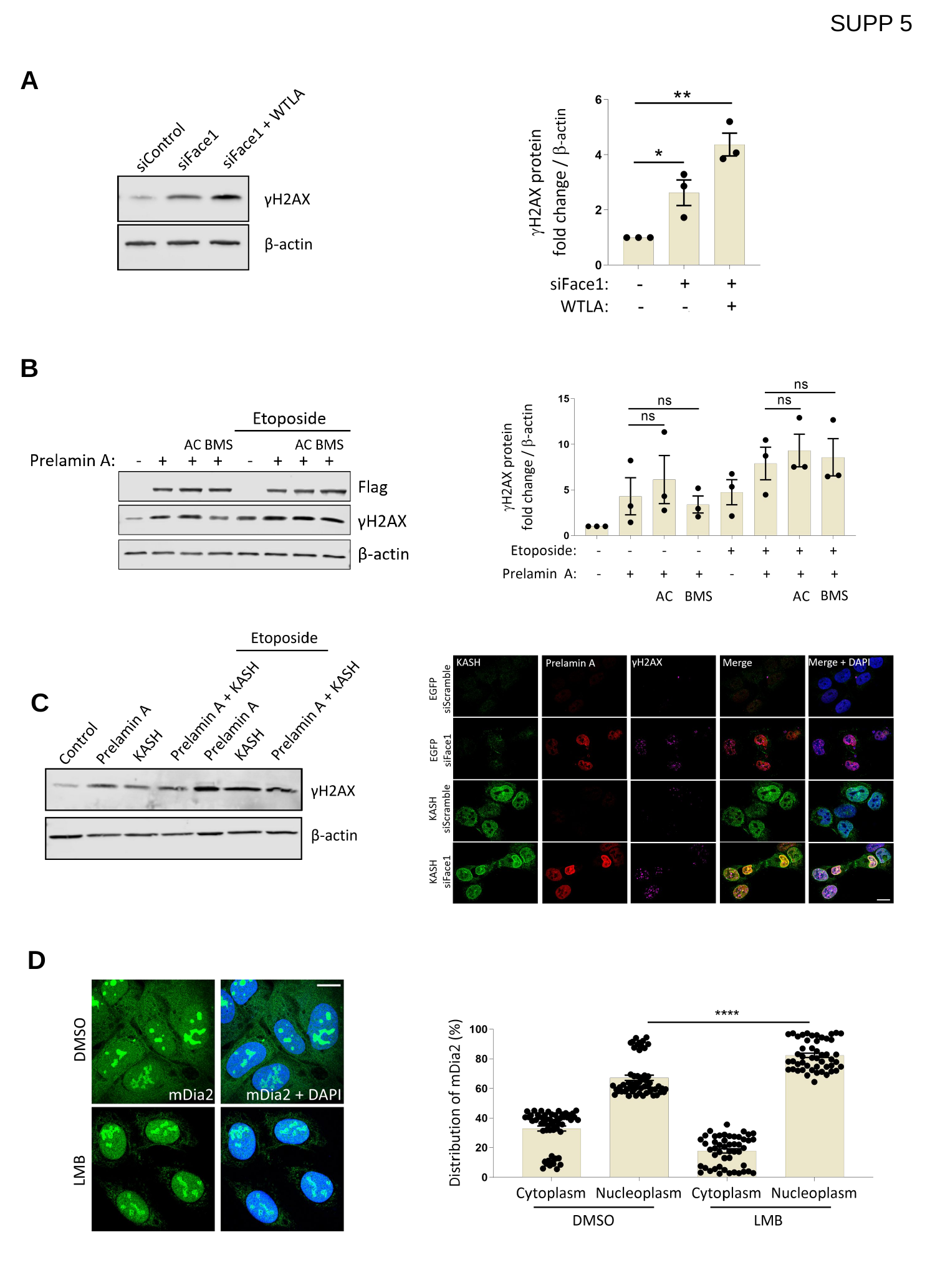

SUPP 5
A
B
C
D

## Slide 2
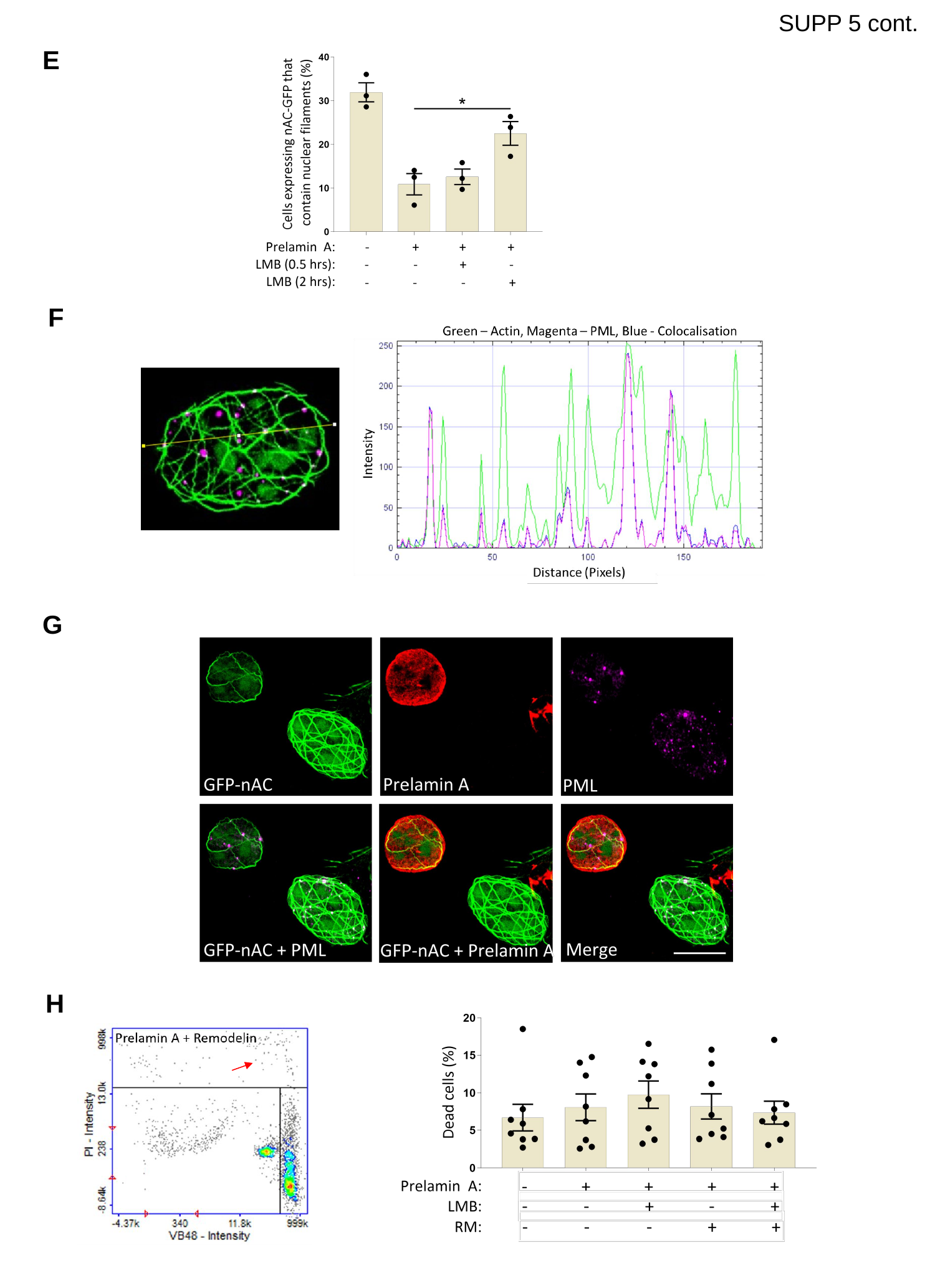

SUPP 5 cont.
E
F
G
H

Supplement: Supplementary file 6 — Supplement Fig 5 [file 41419_2022_5491_MOESM6_ESM.ppt]
