# Supplementary material for: Filamentous nuclear actin regulation of PML NBs during the DNA damage response is deregulated by prelamin A
Source: Cell Death Dis. 2022 Dec 15;13(12):1042. doi: 10.1038/s41419-022-05491-4 (PMC9755150; doi:10.1038/s41419-022-05491-4)
Supplement: Supplementary file 8 — Original Data File [file 41419_2022_5491_MOESM8_ESM.pptx]

## Slide 1
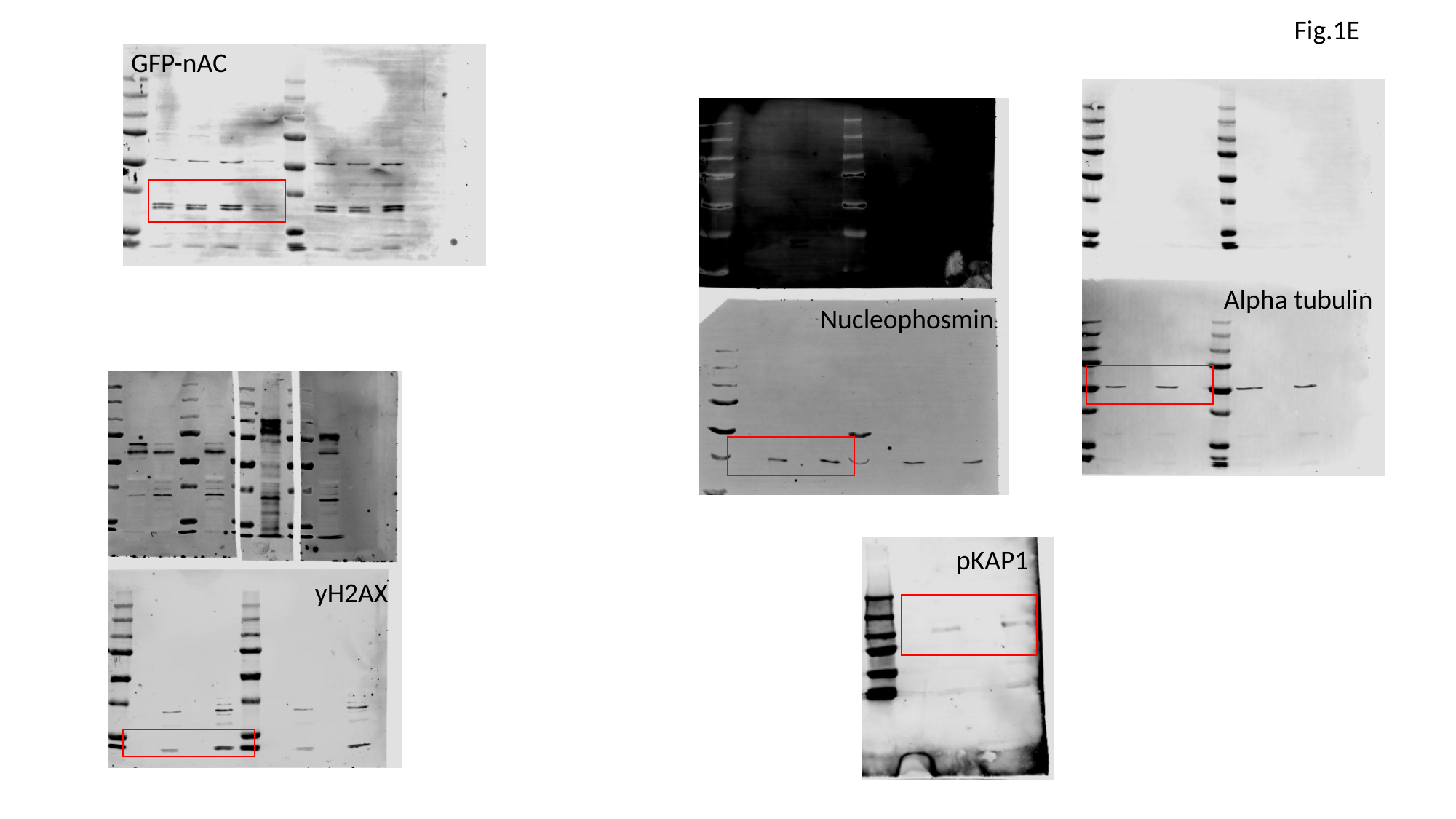

Fig.1E
GFP-nAC
Alpha tubulin
Nucleophosmin
pKAP1
yH2AX

## Slide 2
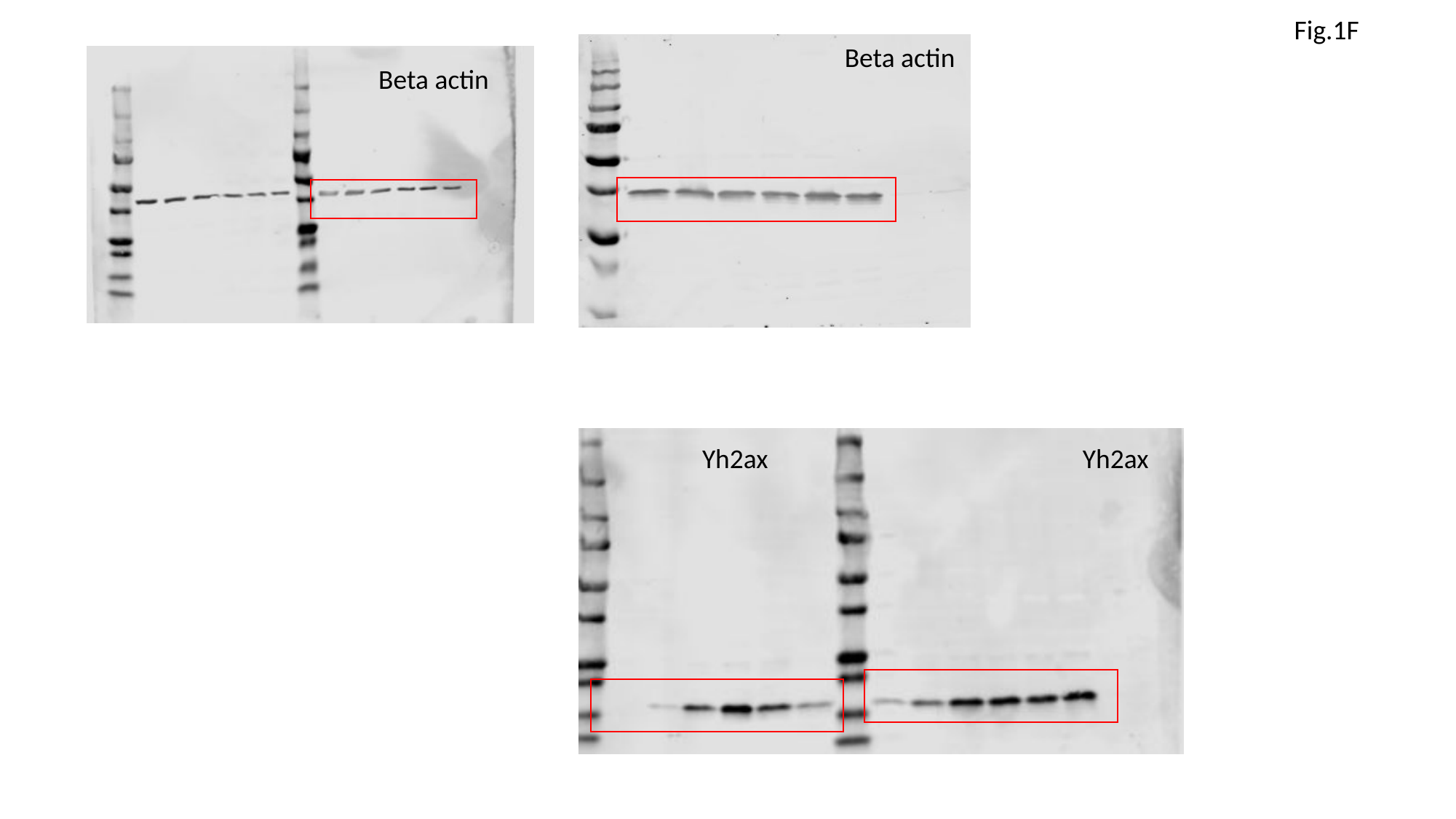

Fig.1F
Beta actin
Beta actin
Yh2ax Yh2ax

## Slide 3
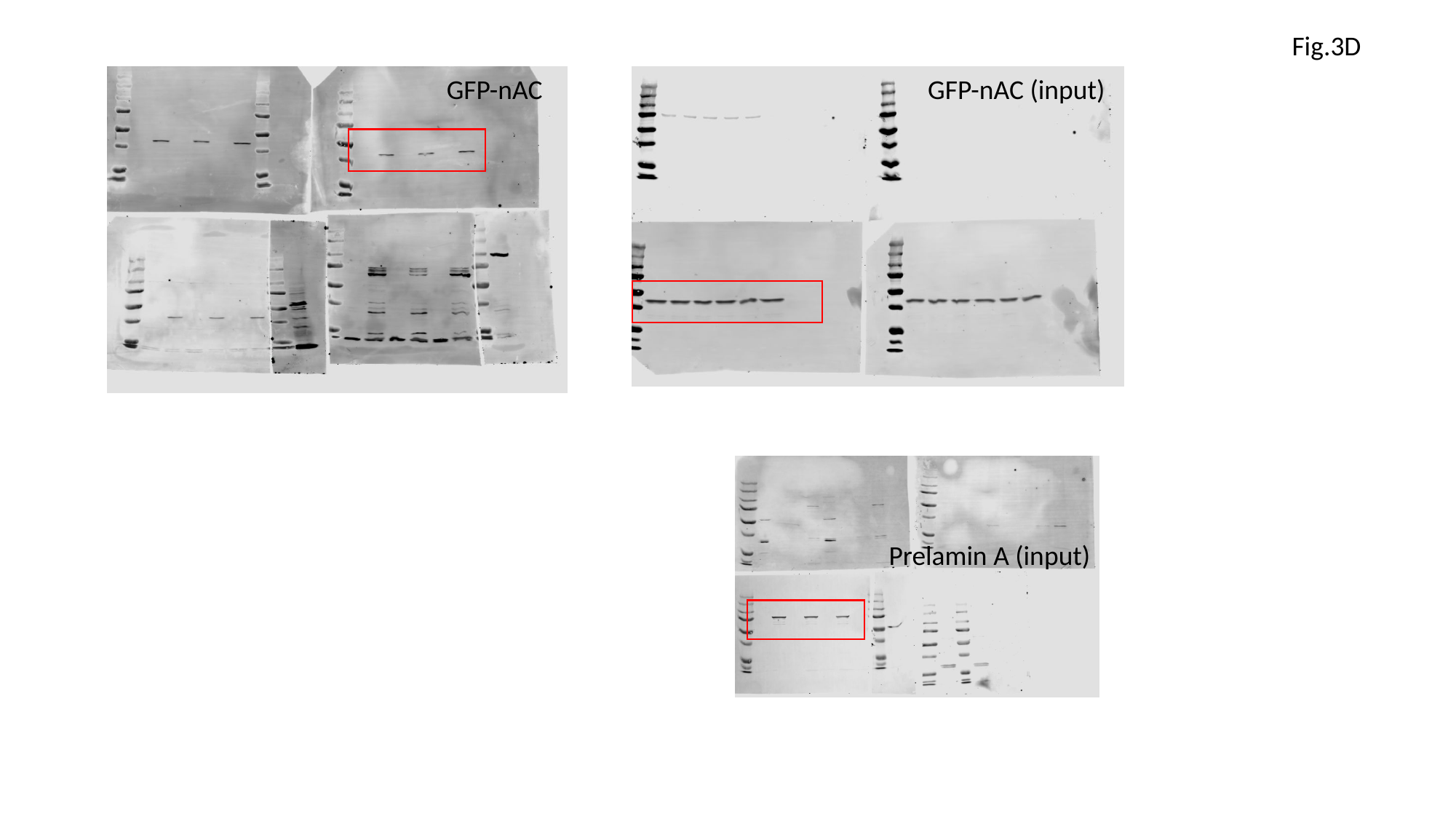

Fig.3D
GFP-nAC
GFP-nAC (input)
Prelamin A (input)

## Slide 4
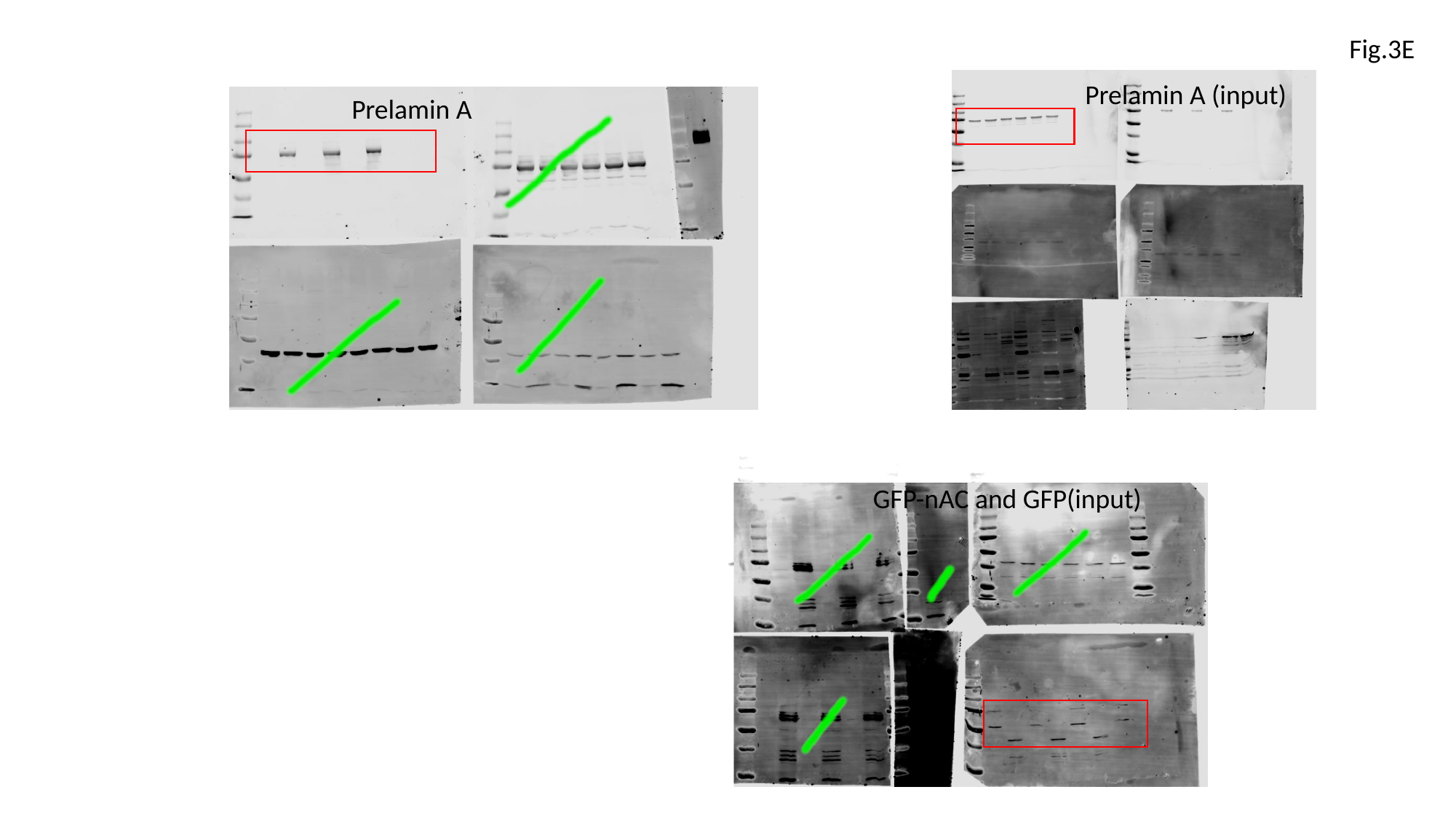

Fig.3E
Prelamin A (input)
Prelamin A
GFP-nAC and GFP(input)

## Slide 5
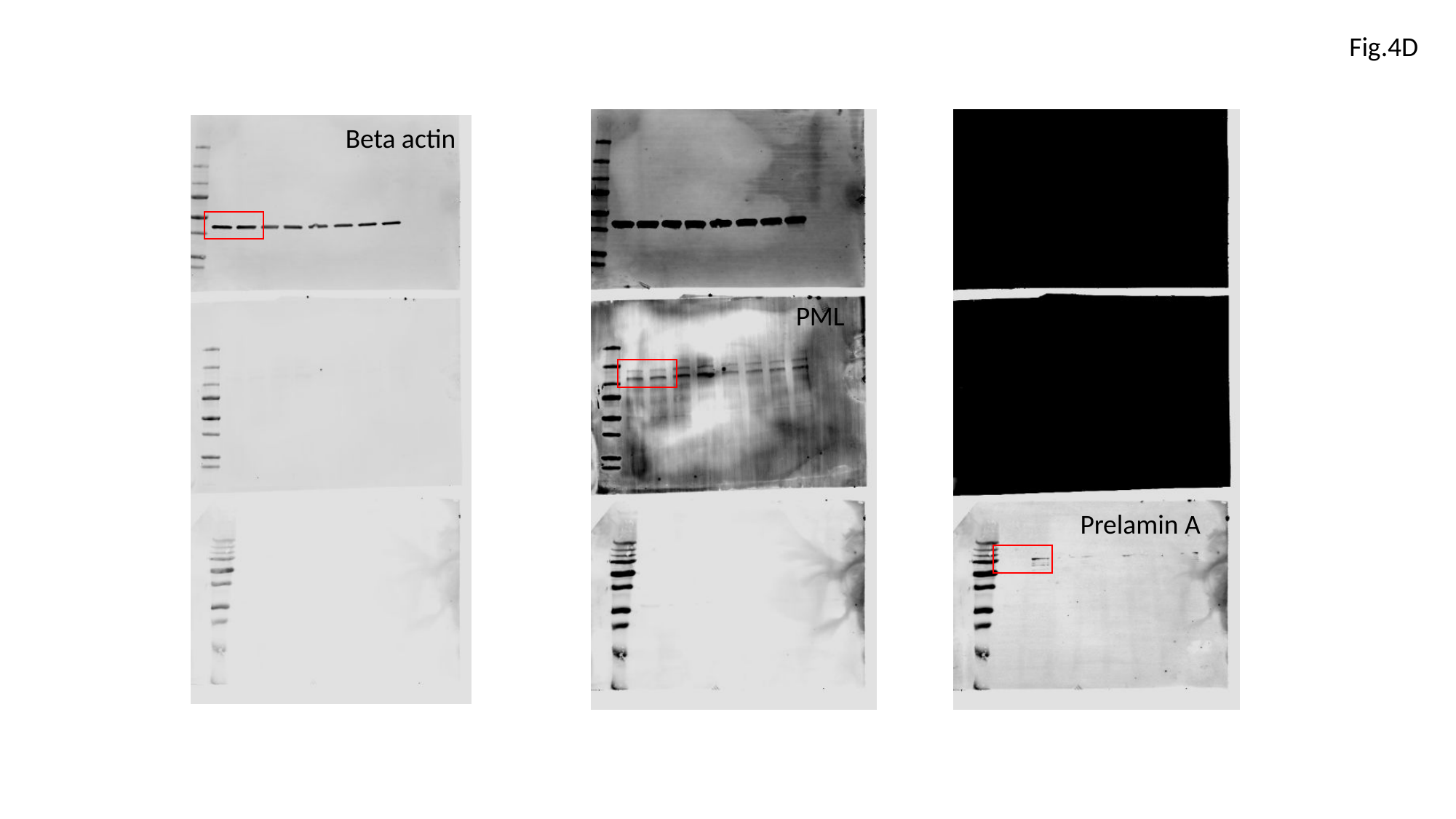

Fig.4D
Beta actin
PML
Prelamin A

## Slide 6
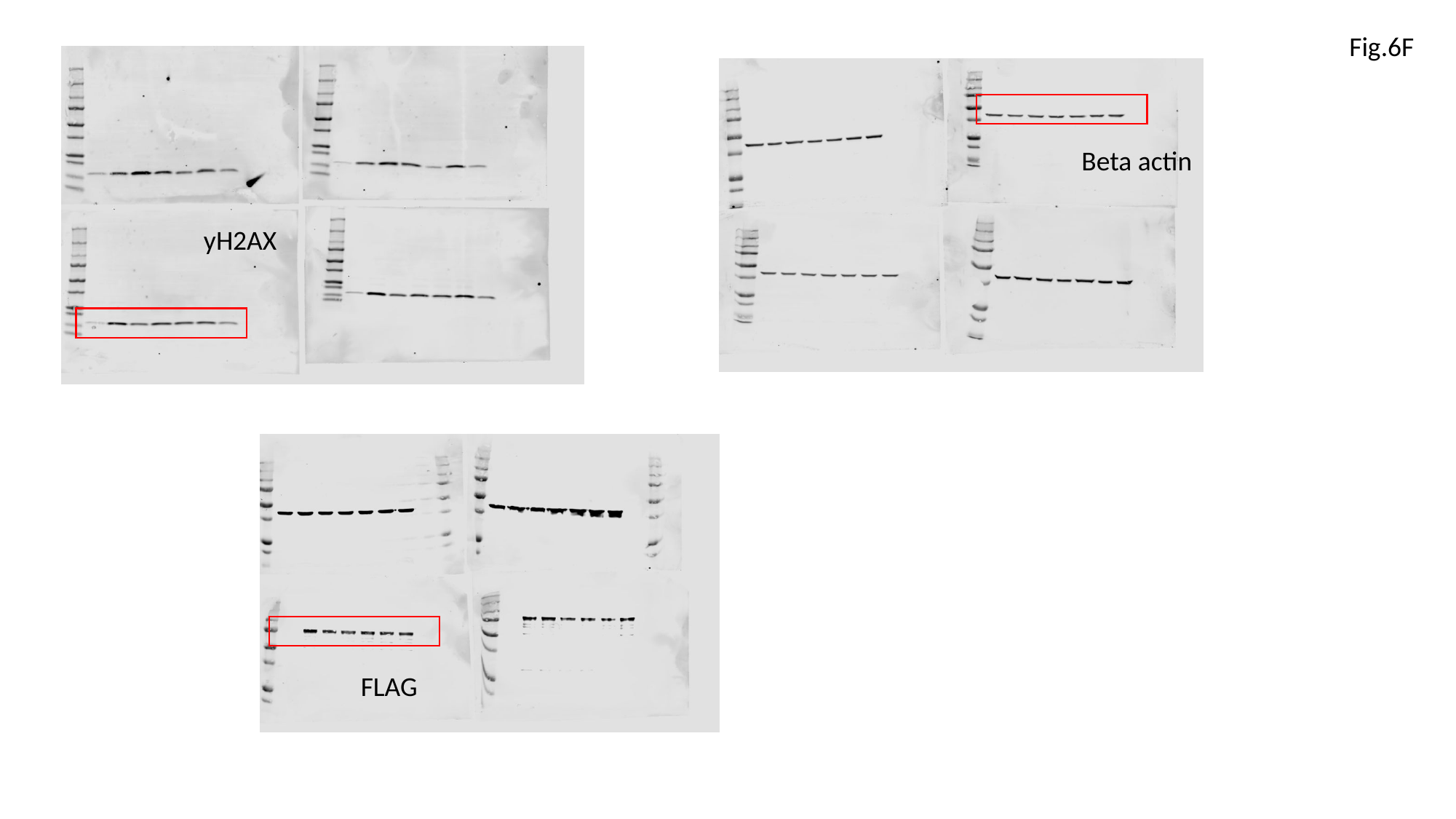

Fig.6F
Beta actin
yH2AX
FLAG

## Slide 7
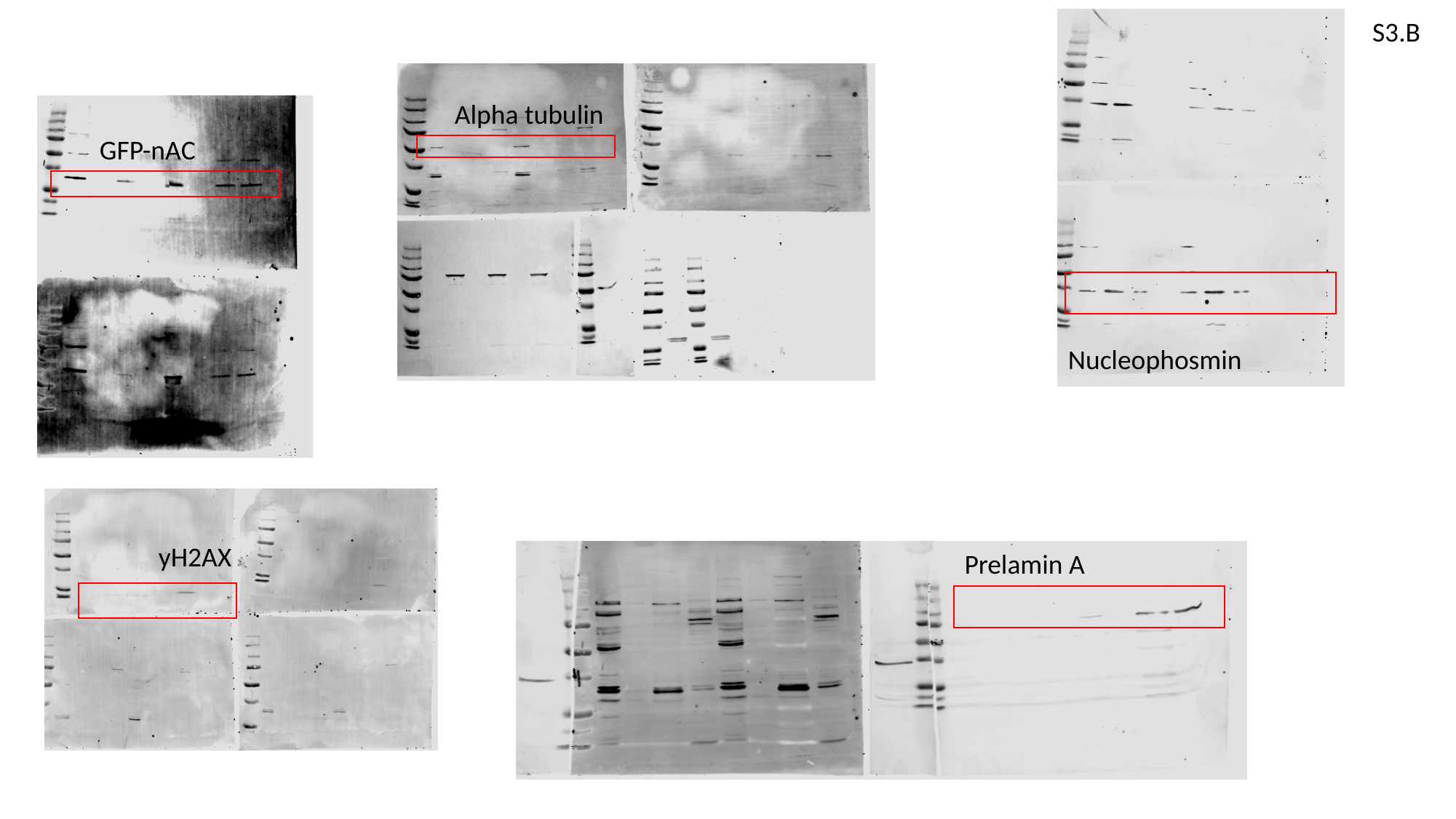

S3.B
Alpha tubulin
GFP-nAC
Nucleophosmin
yH2AX
Prelamin A

## Slide 8
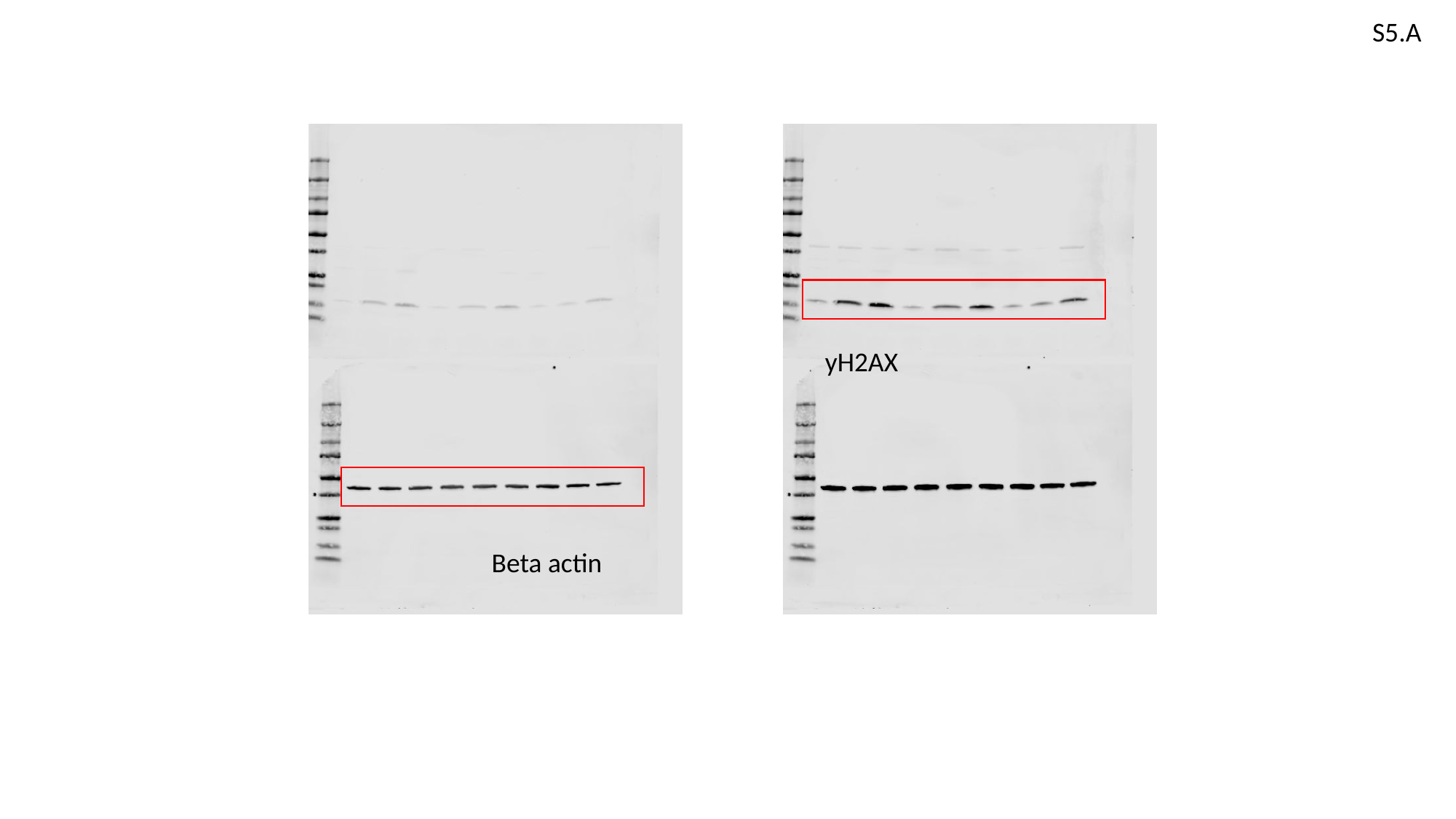

S5.A
yH2AX
Beta actin

## Slide 9
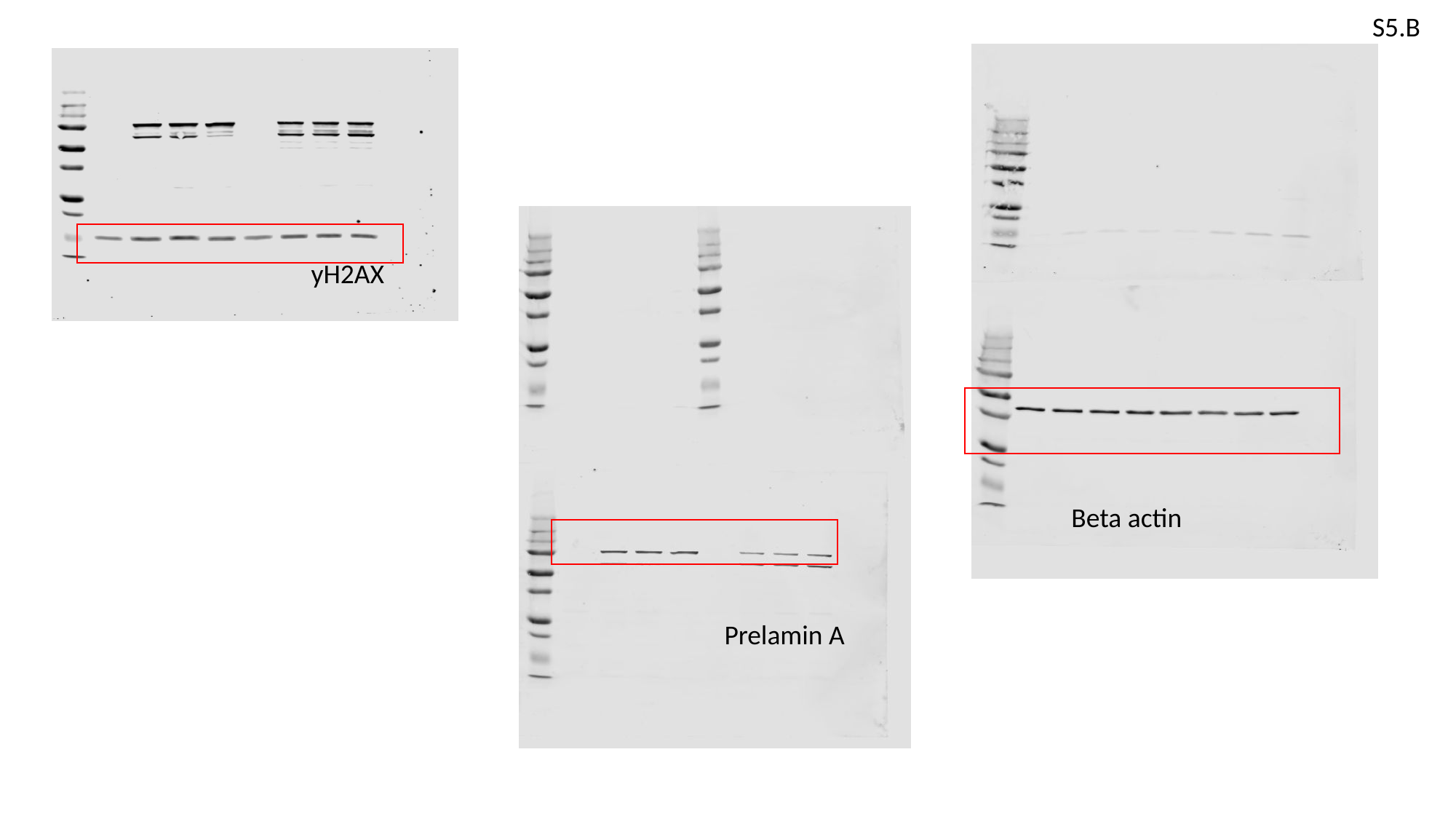

S5.B
yH2AX
Beta actin
Prelamin A

## Slide 10
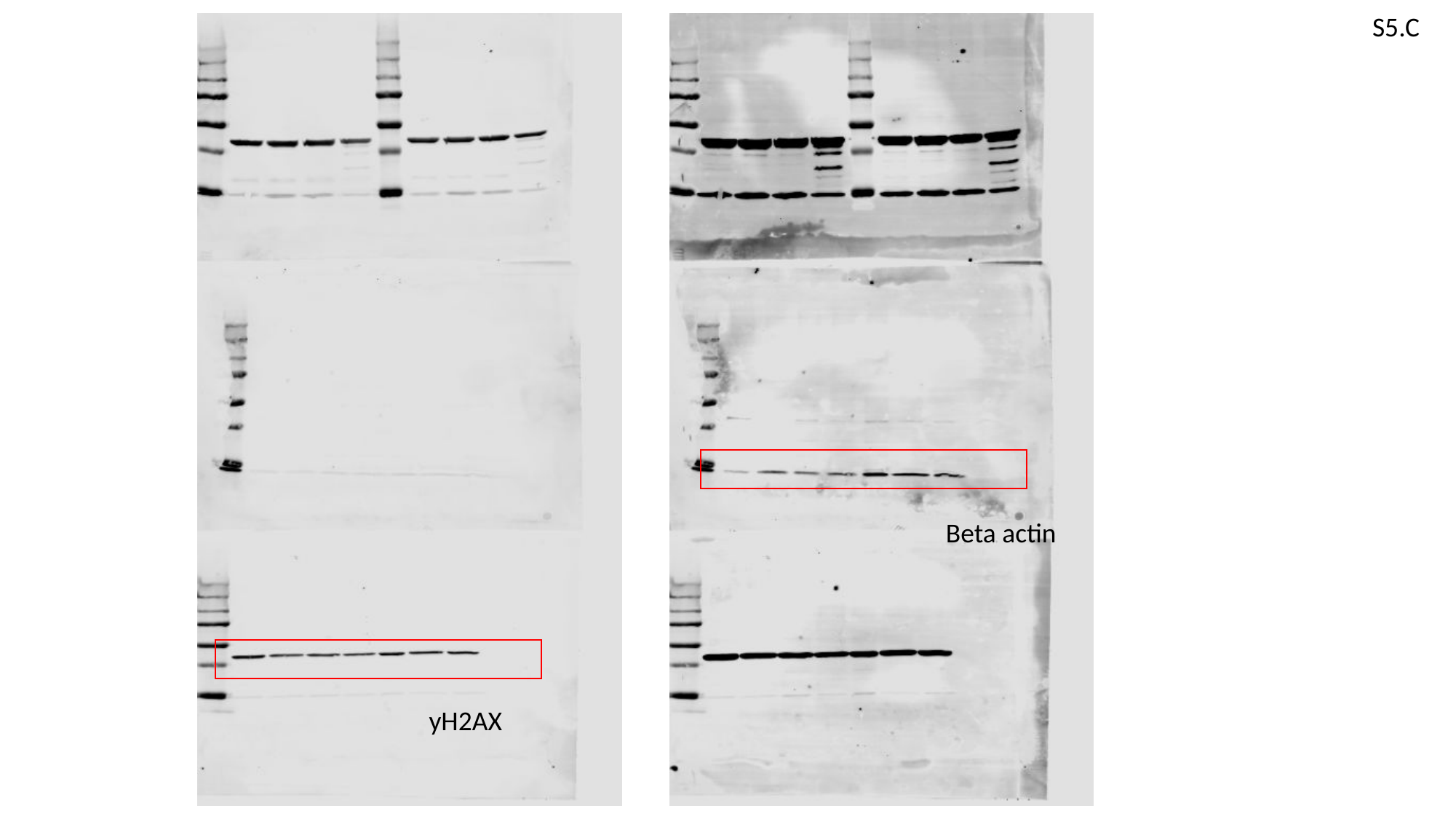

S5.C
Beta actin
yH2AX
