# Supplementary material for: Filamentous nuclear actin regulation of PML NBs during the DNA damage response is deregulated by prelamin A
Source: Cell Death Dis. 2022 Dec 15;13(12):1042. doi: 10.1038/s41419-022-05491-4 (PMC9755150; doi:10.1038/s41419-022-05491-4)
Supplement: Supplementary file 10 — Author contributions as a separate file (this is also included in the main text file) [file 41419_2022_5491_MOESM10_ESM.docx]

AMC and CMS contributed to the conceptualization; AMC, SADS, RH, KS and SU performed experiments; AMC and KS formal analysis; RH, SU, RG and CMS to Resources; AMC and CMS to writing the original draft; AMC, RG and CMS to writing review and editing; CMS to supervision; CMS to project administration; and CMS and RG to funding acquisition.
